# Foreign Mucins Alter the Properties of Reconstituted Gastric Mucus

**DOI:** 10.1021/acs.biomac.4c01629

**Published:** 2025-02-28

**Authors:** Fabio Henkel, Oliver Lieleg

**Affiliations:** † School of Engineering and Design, Department of Materials Engineering, Technical University of Munich, Boltzmannstraße 15, 85748 Garching, Germany; ‡ Center for Protein Assemblies and Munich Institute of Biomedical Engineering, Technical University of Munich, Ernst-Otto-Fischer Str. 8, 85748 Garching, Germany

## Abstract

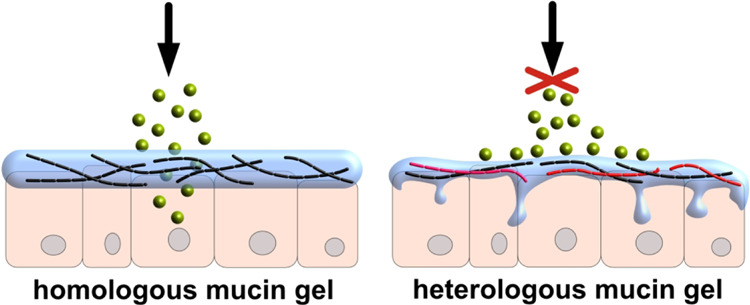

During the course of evolution, distinct mucin subtypes have evolved, that predominantly occur in specific mucus variants of the body. A loss of this clear regional assignment is often associated with pathophysiological conditions such as asthma or gastric cancer. We here reconstitute mucus from different mucin subtypes to elucidate the influence of MUC5B/MUC2 contaminations on physiologically relevant properties of acidic MUC5AC gels as found in the stomach. Our findings indicate that these properties may be critically altered by the presence of an atypical mucin species. A weak integration of a contaminating mucin subtype into the host network yields weak viscoelastic gels with increased barrier capabilities. Unravelling the complex properties of mucosal barriers under disease conditions is crucial for the understanding of mucosal disease progression and for developing drug-carriers to traverse this biological barrier. Here, our results provide useful insights into mechanistic principles governing the physical properties of gastro-intestinal mucus.

## Introduction

Mucus covers all wet epithelial surfaces in mammals. Here, it provides mechanical protection of the underlying tissue by acting as a lubricant, and it serves as the first line of defense by regulating the entry of substances into the body.^1,2^ The highly selective barrier properties of mucus can be attributed to mucins, i.e., large glycoproteins, which form a dense network that allows certain molecules to pass whereas others (depending on their size and charge) are retained by the network.^3^ There are three major variants of secreted mucins, and each of those mucin variants occurs predominantly in a specific region of the body. These mucin variants include MUC5AC (which is usually present in the stomach), MUC5B (which is the predominant mucin subspecies in the lungs), and MUC2 (which occurs in the intestine).^4^

However, this clear regional assignment of those three mucins does not hold true anymore when certain pathological scenarios occur: then, one can observe either a down-regulation of the expression of a region-specific mucin subtype and/or an up-regulation or even *de novo* expression of “foreign” mucin subtypes (*i.e*., of such mucins, that are usually associated with other regions of the body). For instance, the physiological equilibrium between MUC5B and MUC5AC in the lungs can get lost by a downregulation of the predominant MUC5B species (in the case of asthma) or by an upregulation of the MUC5AC variant (which occurs for both, asthma and chronic obstructive pulmonary disease).^5,6^ This imbalance interferes with the mucociliary clearance process^7,8^ and is considered to increase a patient’s susceptibility toward bacterial and viral infections.^8,9^ In the stomach, the *de novo* expression of the lung mucin MUC5B or of the intestinal mucin MUC2 is often associated with bacterial infections^10^ or gastric cancer.^11−13^ However, the interplay of different mucin subspecies is to date not yet well understood.

Interestingly, all secreted mucin variants share a high degree of structural similarity but differ with respect to the sequence of their amino acid backbone.^14,15^ This raises the question why distinct mucin subspecies have evolved and how they differ from each other in terms of their function. Song et al.^7^ found that MUC5AC and MUC5B affect the properties of lung mucus in different ways as MUC5B mobilizes and MUC5AC spatially aligns the ciliary transport of mucus. Furthermore, microrheological measurements conducted in the same study indicate, that MUC5B may reduce micromechanical heterogeneities in MUC5AC gels. Carpenter et al.^16^ investigated the diverse organization and assembly of MUC5AC and MUC5B networks. Their findings suggest that the presence of MUC5B can change the network architecture of a MUC5AC host system. However, it remains unclear how such changes in the network architecture affect the microscopic and macroscopic properties of mucin hydrogels.

In this study, we employ a mucus model system reconstituted from purified mucins to investigate the effect of regionally untypical mucin contaminations on the physiologically relevant properties of a region-specific mucin host system. In particular, we investigate the influence of MUC5B and MUC2 “contaminations” on the mechanical properties and the barrier properties of acidic MUC5AC gels as found in the stomach and show that these physical properties can—depending on the contaminating mucin subtype—critically change. Our findings suggest that the way a contaminating mucin subspecies is integrated into the network architecture decides the fate of the host system: if molecular interactions between MUC5AC and the contaminant are very similar as those between distinct MUC5AC mucins, the contaminant may replace a host mucin without changing the network properties. If this is not the case (as we observe it here for MUC5B contaminations), the MUC5AC host system is turned into a softer macromolecular network with strengthened barrier properties, and the latter probably result from an increased chain mobility within the mixed mucin network.

## Materials and Methods

### Purification of Mucins

MUC5AC and MUC2 were purified from porcine stomachs and porcine small intestines, respectively. Both organ types were freshly obtained from a local butcher (Metzgerei Mundlhof, Allershausen, Germany).

MUC5AC was purified according to a modified version of the protocol described by Marczynski et al.^17^ In brief, gastric mucus was harvested from pig stomachs, diluted with 1× phosphate buffered saline (PBS, 3 parts PBS/1 part raw mucus) and subsequently supplemented with 0.01% (w/v) sodium azide (Carl Roth, Karlsruhe, Germany) to prevent bacterial growth. The mixture was homogenized at 4 °C overnight under slight steering followed by serial filtration using three different metal grid filters (500, 200, and 125 μm). The filtrate was collected and further purified using size exclusion chromatography (SEC, Äkta Go, Cytiva, Marlborough). Finally, the purified MUC5AC solution was desalted using a crossflow (Cytiva, hollow fiber cartridge, 300 kDa molecular weight cutoff (MWCO)), lyophilized and stored at −70 °C until further use.

MUC2 was purified from intestinal mucus according to a modified version of the protocol described by Schömig et al.^18^ Intestinal mucus comprises two mucosal layers: a firmly surface bound, insoluble layer and a loosely bound, soluble layer.^19^ The loosely bound, soluble fraction of the intestinal mucus was flushed out from approximately 5 m of porcine small intestinal tissue using 1× PBS. To precipitate any remaining parts of the insoluble mucus fraction, 2 M guanidine hydrochloride (Carl Roth) was added to the solution. Additionally, the solution was supplemented with 1 M sodium chloride (Carl Roth) to weaken electrostatic interactions between the MUC2 molecules and potential impurities as well as with 50 mM ethylenediaminetetraacetic acid (EDTA, Carl Roth) to prevent protease activity and with 0.01% (w/v) sodium azide. As described for the purification of MUC5AC, the raw mucus solution was homogenized overnight, followed by ultracentrifugation at 45.000 rpm for 30 min (LE-70 ultracentrifuge, Beckmann Coulter, Brea, USA; type 45 Ti fixed angle rotor, Beckmann Coulter) at 4 °C. The supernatant was collected and further purified using SEC, desalted using a crossflow, lyophilized, and finally stored at −70 °C as described above.

Bovine submaxillary mucin (BSM, MUC5B) was purchased from Sigma-Aldrich (St. Louis) and repurified by dissolving 100 mg of BSM in 100 mL 1× PBS containing 1 M sodium chloride. Then, the solution was subjected to SEC, desalted using a crossflow (filter: 100 kDa MWCO), lyophilized, and finally stored at −70 °C.

### Gel Electrophoresis

Sodium dodecyl sulfate (SDS)-polyacrylamide gel electrophoresis (PAGE) was employed to assess the purity of the mucin samples. The mucin samples were prepared by mixing a 1% (w/v) aqueous mucin solution (MUC5AC, MUC2, repurified MUC5B, or commercial BSM) with 4× mPAGE running buffer (Sigma-Aldrich, mixing ratio 1:1). A 10% Bis-Tris mPAGE mini gel (12-well, Sigma-Aldrich) was installed in a Mini-Protean 3 electrophoretic chamber (Bio-Rad Laboratories Inc., Hercules) and loaded with 10 μL of each of the mucin samples. The first gel pocket was loaded with 5 μL mPAGE Color Protein Standard (Sigma-Aldrich) for molecular weight comparison. Subsequently, the gel was run at 200 V for 30 min in 3-morpholinopropanesulfonic acid (MOPS, pH 7.7) running buffer containing 2.5 mM MOPS (AppliChem, Darmstadt, Germany), 2.5 mM tris(hydroxymethyl)aminomethane (TRIS) base (AppliChem), 0.05 mM ethylenediaminetetraacetic acid (EDTA, Carl Roth), and 0.005% (w/v) SDS (Carl Roth). After completion of the electrophoresis run, the gel was removed from the gel cassette, transferred into a glass staining container, and the protein bands were visualized using a Pierce Silver Stain Kit (Thermo Fisher Scientific, Waltham).

### Turbidimetric Measurements and Phase Contrast Imaging

Stock solutions of MUC5AC, MUC5B, and MUC2 were prepared in ultrapure water at a concentration of 20 mg/mL each. Mixed mucin samples were prepared from these stock solutions by combining three parts of a MUC5AC host solution with one part of a contaminating mucin solution (either MUC5B or MUC2). Subsequently, the mixed mucin samples were diluted at a ratio of 1:1 with 20 mM sodium acetate buffer at a desired pH level (pH 3 or pH 4). A MUC5B host system comprising MUC5AC as contaminating mucin subspecies was prepared at neutral pH by mixing three parts of a 1% (w/v) MUC5B stock solution in Dulbecco’s phosphate buffered saline (D-PBS) with one part of a 1% (w/v) MUC5AC solution in D-PBS. The turbidities of the freshly prepared mucin mixtures were measured every 30 min (for a total time of 2 h) in a UV-transparent 96-well-plate at a wavelength of 300 nm using a plate reader (Varioscan Lux, Thermo Fisher Scientific) and a sample volume of 75 μL each. For each mixture, control groups comprising the individual components of the mixture at their respective concentration, i.e., 0.75% (w/v) and 0.25% (w/v), were measured in the same manner. For the control groups, the missing component of the mixed mucin samples was replaced with ultrapure water or D-PBS, respectively. Additionally, the turbidity of pure mucin samples was measured at a concentration of 1% (w/v) as a reference.

After the turbidimetric measurements, phase contrast images of all samples were obtained using a Leica DMi 8 inverse microscope (Leica Microsystems GmbH, Wetzlar, Germany) and a 4× objective (Universal 4×/0.10 DRY).

### Rheological Measurements

MUC5AC, MUC5B, and MUC2 were separately dissolved in nine parts of ultrapure water, then the macromolecular concentration was adjusted to 20 mg/mL by adding one part of 100 mM sodium acetate buffer at a desired pH (pH 3 or pH 4). Mixed mucin samples were prepared by mixing three parts of the previously prepared MUC5AC solution with one part of either the MUC5B solution or the MUC2 solution before adding the buffer solution. All samples were first equilibrated for 2 h at room temperature (RT). Subsequently, the storage modulus and loss modulus of the pure mucin samples were compared to those of the mixed mucin samples across a frequency spectrum of 0.1–10 Hz. Additionally, the rheological properties of a pure MUC5AC sample supplemented with ultrapure water instead of a contaminating mucin-subtype was determined as a control group. All measurements were performed on a research-grade shear rheometer (MCR 302, Anton-Paar, Graz, Austria) using a temperature controlled bottom plate (P-PTD200/56, Anton-Paar, *T* = 20 °C), a planar measuring head (PP25, Anton-Paar), and a sample volume of 100 μL. Since mucin hydrogels are often fragile and may be disrupted by high shear forces, the front ends of any pipet tips used to transfer the mucin samples to the bottom plate of the rheometer were cut off to enlarge their openings. For all rheological measurements, due care was taken not to exceed the linear regime of the viscoelastic material response. For that purpose, each sample was subjected to a pretest assessing the material strain at a minimum applicable stress in a torque controlled operating mode of the rheometer (employing a torque of *M* = 0.5 μNm). The such obtained strain value was then multiplied by a factor of 1.5 and used as a target strain for the subsequent rheological measurement in a strain controlled operating mode.

Creep tests were conducted using the same setup and sample preparation as described above. Each sample was subjected to a constant torque of 1 μNm. The load was maintained for 200 s while monitoring the creep compliance *J*(*t*) as calculated by the devices based on the ratio of the applied stress and the observed strain. The almost linear behavior of the creep compliance in the last 50 s of the measurement was then approximated using a linear regression model (Prism 10, version 10.4.0, GraphPad Software, La Jolla).

### Molecular Translocation Assay

The diffusive translocation of differently charged molecules across a MUC5AC gel contaminated with either MUC5B or MUC2 was tested following a modified version of the protocol described in Marczynski et al.^20^ For that purpose, an aqueous solution of MUC5AC supplemented with MUC5B or MUC2 (mixing ratio 3:1) was prepared at a total macromolecular concentration of 2% (w/v). Subsequently, 50 μL of the prepared mucin solution were pipetted into 24-well cell culture inserts (CellQuart, Sabeu GmbH & Co., KG, Northeim, Germany). The bottom membrane of these cell culture inserts (pore size: 0.4 μm) retained the mucins but allowed smaller dextran molecules (see below) to pass. To trigger acidic gelation of the mucin solution *in situ*, the filled cell culture inserts were immersed in 10 mM sodium acetate buffer (prepared at pH 3 or pH 4) in a 24-well plate for 2 h. Next, 5% (w/v) fluorescently labeled test molecules of different charge with an average molecular weight of approximately 4 kDa were separately dissolved using the same buffer. Those test molecules comprised: dextran (4 kDa, uncharged), Diethylaminoethyl (DEAE)-dextran (3–6 kDa, cationic) and Carboxymethyl (CM)-dextran (4 kDa, anionic); all dextran variants carried a fluorescein isothiocyanate (FITC) label and were purchased from Sigma-Aldrich (St. Louis). Then, the diffusive translocation of these test molecules was tested by placing a 5 μL droplet of a dextran solution on top of a mucin gel. After 1 h of incubation at RT on a slowly moving tilting shaker (Everlast Rocker 247, Benchmark Scientific, Sayreville), 140 μL aliquots of the buffer solution were removed from each well, and the amount of test molecules that had traversed the mucin gel was quantified by measuring the fluorescence signal at an excitation wavelength of 498 nm and an emission wavelength of 517 nm using a plate reader (Varioscan Lux). Additionally, the translocation behavior of the dextrans across pure MUC5AC, MUC5B, and MUC2 samples reconstituted at the same macromolecular concentration as the mixed mucin gel was determined in in a similar manner. Finally, the data obtained for all variants of mucin gels was normalized to the data obtained for the respective pure MUC5AC gels.

### ζ-Potential Measurements

MUC5AC, MUC5B, and MUC2 were separately dissolved in ultrapure water at a concentration of 0.1% (w/v). Next, the MUC5AC solution was mixed with either the MUC5B solution or the MUC2 solution at a ratio of 3:1 and subsequently diluted 1:1 with 10 mM sodium acetate buffer at pH 3 or pH 4, depending on the particular sample. Additionally, pure mucin samples were prepared from the stock solutions at the same macromolecular concentration as the mixed mucin samples. The ζ-potentials of all samples were measured with a Litesizer 500 (Anton-Paar) and an Omega cuvette (Anton-Paar) at a target temperature of 25 °C by means of electrophoretic light scattering using a Smoluchowski approximation.^21−23^

### Sequence Alignment and Structure Prediction

The amino acid sequences of MUC5AC, MUC5B, and MUC2 were obtained from the Uniprot database (see Supporting Information, section 1). For all further analyses, only the first 1100 amino acids of each amino acid sequence (those represent the N-terminal regions of the three different mucin variants) were used. These sequences were aligned and analyzed for similarities with the Basic Local Alignment Search Tool (BLAST) for proteins provided by the National Library of Medicine (blastp suite, National Library of Medicine, Rockville Pike).

The folding structures of the N-terminal regions were predicted using the software AlphaFold 3.^24^ Structure predictions were made for N-terminal complexes comprising two and four termini, respectively; herein, each complex comprised one or more MUC5AC termini as a host system and one MUC5AC, MUC5B, or MUC2 terminus as an additional interaction partner. The structure predictions were visualized with PyMol (The PyMOL Molecular Graphics System, Version 3.1.1, Schrödinger, New York).

### Data Analysis and Statistics

If not stated otherwise, all statistical tests were performed using the software Prism 10. First, a Shapiro–Wilk test was performed to assess the normal data distribution in the tested sample groups. Next, the sample groups were tested for homoscedasticity using a Levene’s test implemented in Matlab (R2021, MathWorks, Natick). Depending on the outcome of these pretests, a suitable version of a two-sample test was chosen to test for statistically significant differences between the sample groups based on a minimum tolerated error probability of *p* < 0.05. If the data was determined to be normally distributed and homoscedastic, a parametric *t* test (with Welch’s correction in case of heteroscedasticity) was employed. If the condition of normal distribution was not met, a Mann–Whitney test was conducted as an alternative.

## Results and Discussion

**Figure 1:**
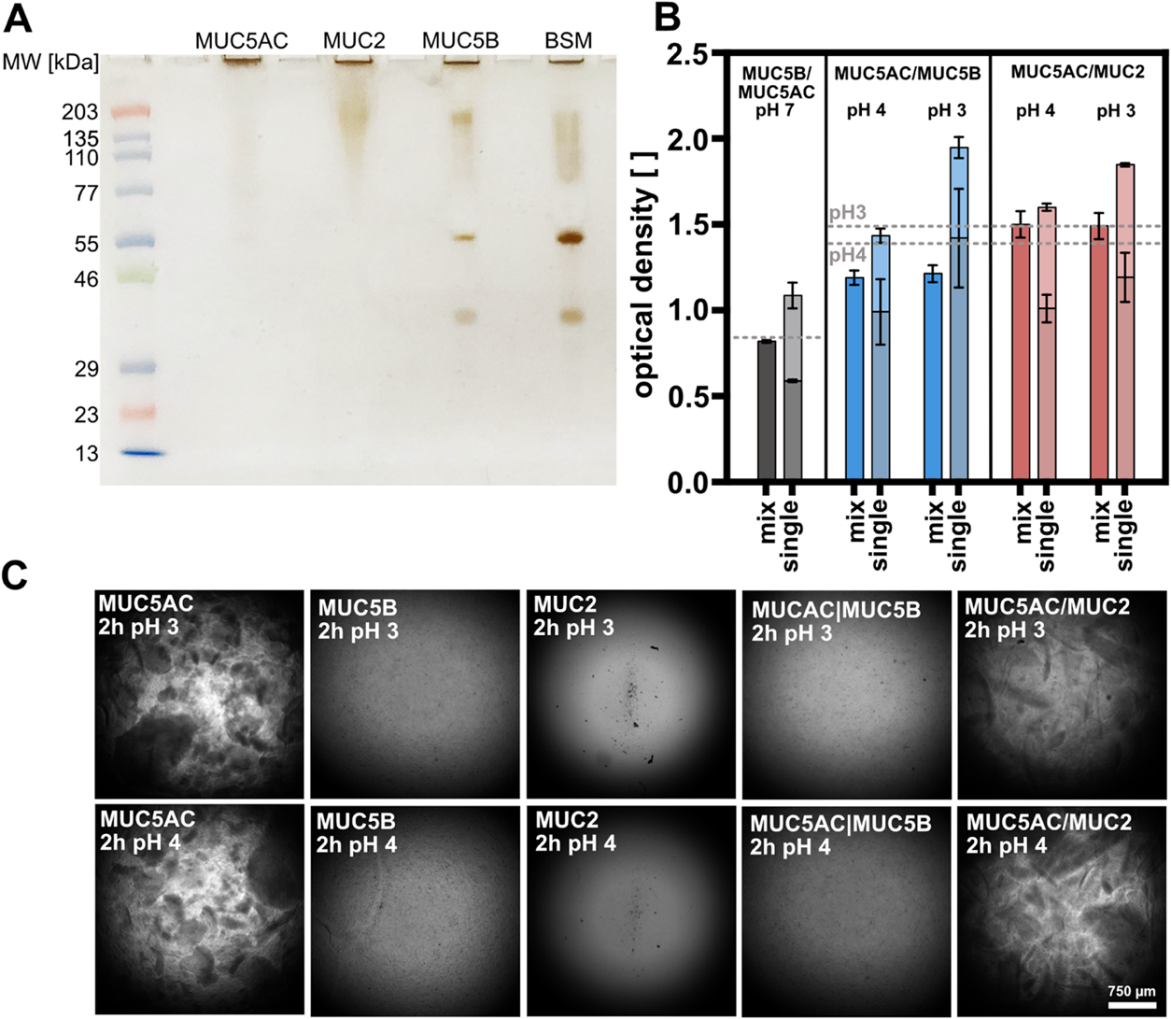
Purity and turbidity of pure and mixed mucin samples. SDS-PAGE of purified MUC5AC, MUC2 and MUC5B samples and off-the-shelf purity of commercial BSM (A); measured turbidity of binary mixtures of mucin subtypes (full bars) compared to their expected turbidity according to the sum of their individual components (stacked bars; lower bar: host system; upper bar: contaminating mucin subtype) after 2 h of equilibration, dashed lines indicate the optical densities of the host system at a concentration of 1% (w/v) (*n* = 3) (B); phase contrast images of the individual components and corresponding mixtures after 2 h (C).

The use of highly pure mucins is crucial to study the interplay of different mucin subtypes. An SDS-PAGE conducted with our purified MUC5AC and MUC2 mucin variants as well as with MUC5B repurified from commercial BSM was treated with a highly sensitive silver staining and confirms the purity of the different mucins used in this study. As can be seen from Figure 1A, owing to their high molecular weight of several MDa,^25^ the mucin macromolecules cannot enter the gel and are trapped inside the gel pockets. The lab purified mucin variants MUC5AC and MUC2 contain hardly any low molecular weight impurities. In contrast, the commercial MUC5B variant (BSM) shows bands at 55 and ∼40 kDa, which points at small amounts of low molecular weight impurities. However, these are reduced by our repurification of the commercial BSM as indicated by the reduced intensity of these bands. A similar gel stained with a conventional, but less sensitive Coomassie staining returns a similar result (see Supporting Information, section 2). Furthermore, an evaluation of the hydrodynamic size of the mucins at neutral pH indicates that the purified mucins do not contain big macromolecular aggregates (see Supporting Information, section 3).

To investigate how contaminations of an atypical mucin subtype affect a mucin host system we first compare the turbidity of different mucin/mucin mixtures to the sum of the turbidity of their individual components. This approach is motivated by previous results, which demonstrated that turbidimetric measurements can be indicative of structural changes within mucin networks.^20^ According to Beer’s law, for a macromolecular solution comprising two macromolecular components, the optical density of a homogeneously mixed system should equal the sum of the optical densities of its components.^26,27^ A mismatch between this expected value and the observed optical density of the mixed system points toward structural differences in the network architecture of the mixture compared to the network architecture of the individual components. More specifically, for mixtures created from different mucin subtypes, we expect that local heterogeneities in the sample (such as a local phase separation of the two mucin types or local changes in the macromolecular density resulting from, *e.g*., an aggregation of the mucins) may result in samples that scatter light more strongly than the individual macromolecules combined.

Figure 1B gives an overview over the turbidity of binary mucin mixtures generated from different mucin subtypes in comparison to their expected turbidity according to Beer’s law (the latter is indicated by the stacked bars, wherein the lower bar represents the optical density of the part forming the host system and the upper bar represents the optical density of the contaminating part). Interestingly, for all mucin mixtures studied here, we find that the turbidity of the mixed mucin system is reduced compared to the result obtained for the sum of the respective components (see Figure 1B). This mismatch between the expected and the measured optical density is more pronounced when analyzing systems reconstituted at a lower pH and when studying the samples after longer equilibration times (see Supporting Information, section 4).

Different from our initial expectation, we find lower (and not higher) optical densities in the mixed systems than expected. For instance, for the MUC5B host system containing MUC5AC as a contamination (the “MUC5B/MUC5AC” sample), the optical density measured after 2 h is ∼25% lower than what could be expected based on the individual contributions of MUC5B and MUC5AC, respectively. This suggests that heterogeneities occurring in the pure mucin network are reduced in the mixed system. Indeed, such a homogenization of the mixed MUC5B/MUC5AC system (at neutral pH) compared to the pure mucin systems is in line with observations by Song et al.:^7^ there, single particle tracking experiments were conducted with an airway mucus model system and demonstrated that heterogeneities in the network architecture of the MUC5AC component are reduced in the presence of MUC5B. Such MUC5AC contaminations of a MUC5B host system are physiologically relevant as they can occur in the human lungs, and these particular mixtures have already been studied.^7,8,16^ Thus, we here focus on the effect of mucin (MUC5B or MUC2) contaminations on MUC5AC host systems at acid pH levels, which have not been characterized yet. Those mucin mixtures are highly relevant as well since a *de novo* expression of MUC5B or MUC2 in an acidic MUC5AC host environment (which is present in the healthy stomach) is associated with pathophysiological conditions such as gastric cancer^28,29^ or bacterial infections caused by, *e.g*., *Heliobacter pylori*.^10^

For this MUC5AC host system, a reduction in turbidity predominately occurs in the presence of MUC5B as a contaminating mucin-subtype. Here, after an equilibration time of 2 h, we find optical densities that are reduced (compared to the expected value) by ∼17% (pH 4) and ∼38% (pH 3), respectively. Interestingly, this effect is less pronounced for mixtures of MUC5AC and MUC2: there, it mainly occurs at pH 3 (reduction of ∼19%) but is nearly absent at pH 4. It is worth mentioning that, throughout all sample groups, the measured optical densities of the mixtures remain constant over time, whereas the expected optical densities increase over time (see Supporting Information, section 4). At this point, it is important to recall that said expected optical density values constitute the sum of the optical densities of the individual components, and that the microstructure of systems created from these individual components may change over time, *e.g.*, if mucin aggregation occurs (for instance, as part of the pH-induced gelation of mucin networks). Thus, it seems that the contaminating mucin subtype has a homogenizing effect on the MUC5AC host system by reducing the aggregation propensity of the MUC5AC oligomers. And indeed, phase contrast images acquired for mixed and pure mucin systems (Figure 1C) support this notion. Furthermore, also an evaluation of the hydrodynamic size of mucins at low macromolecular concentrations indicates that mixtures of MUC5AC and either MUC5B or MUC2 tend to form smaller aggregates than observed for pure MUC5AC samples at acidic pH-levels (see Supporting Information, section 3).

These results point toward structural changes of the MUC5AC host system in the presence of site-unspecific mucin contaminations at a macromolecular mass ratio of 3:1. To the best of our knowledge, there is currently no data on pathophysiologically relevant mass ratios of MUC5AC to MUC5B or MUC2 available in the literature. This may be attributed to the specific experimental techniques used to assess the composition of gastric mucus. These techniques usually rely on an immunohistochemical assessment of tissue samples.^10,12,28^ Here, different mucin subtypes are detected using antibody staining and subsequent microscopy imaging of the tissue samples, which allows for a qualitative assessment of the detected mucin subtypes but makes it difficult to quantify the amount of these subtypes. Furthermore, histological samples are usually obtained from small, thin tissue slices, which provide only a very localized picture of the overall tissue. Even for a single type of disease such as gastric cancer, the *de novo* expression of site-unspecific mucins is not consistently observed throughout a cohort of patients, and the site of expression within the stomach can vary between patients.^12^ Taken together, this suggests, that the *de novo* expression of site-unspecific mucins in the stomach may be subject to a high level of biological variability. To address this variability, we conduct turbidimetric measurements of MUC5AC samples contaminated with MUC5B at different ratios (see Supporting Information, section 5). Here, a reduced optical density of the mixed mucin samples is observed across all mixing ratios, but is most pronounced when mixing 75% of MUC5AC and 25% of MUC5B (3:1 ratio). Hence, this ratio is used for all further experiments. However, these results suggest that the observed effects of foreign mucin contaminations on a MUC5AC host system are quite robust to changes in the concentration of the contaminating mucin subtype. Thus, the effects of foreign mucin types, which we here mechanistically study for a model contamination ratio of (3:1), may apply to a wide range of (pathophysiologically relevant) concentrations.

MUC5AC mucins occur in the stomach, where—triggered by the acidic environment—they assemble into a viscoelastic hydrogel that constitutes the structural and functional component of the mucosal layer lining the inner epithelial surface of the stomach. This mucosal layer protects the underlining epithelium against the acidic gastric juice and mechanical damages.^30^ Furthermore, it acts as the first line of defense by providing a highly selective barrier that prevents the invasion of, e.g., toxic molecules or pathogenic bacteria into the underlying tissue.^3,31^ Both functions critically depend on the architecture of the mucin network on a microscale. However, microstructural changes of the mucin network as observed for MUC5AC/MUC5B systems at pH 3 and pH 4 as well as for MUC5AC/MUC2 systems at pH 3 can be sufficient to affect the macroscopic properties of the mucin barrier and thus may interfere with its physiological functions.

**Figure 2:**
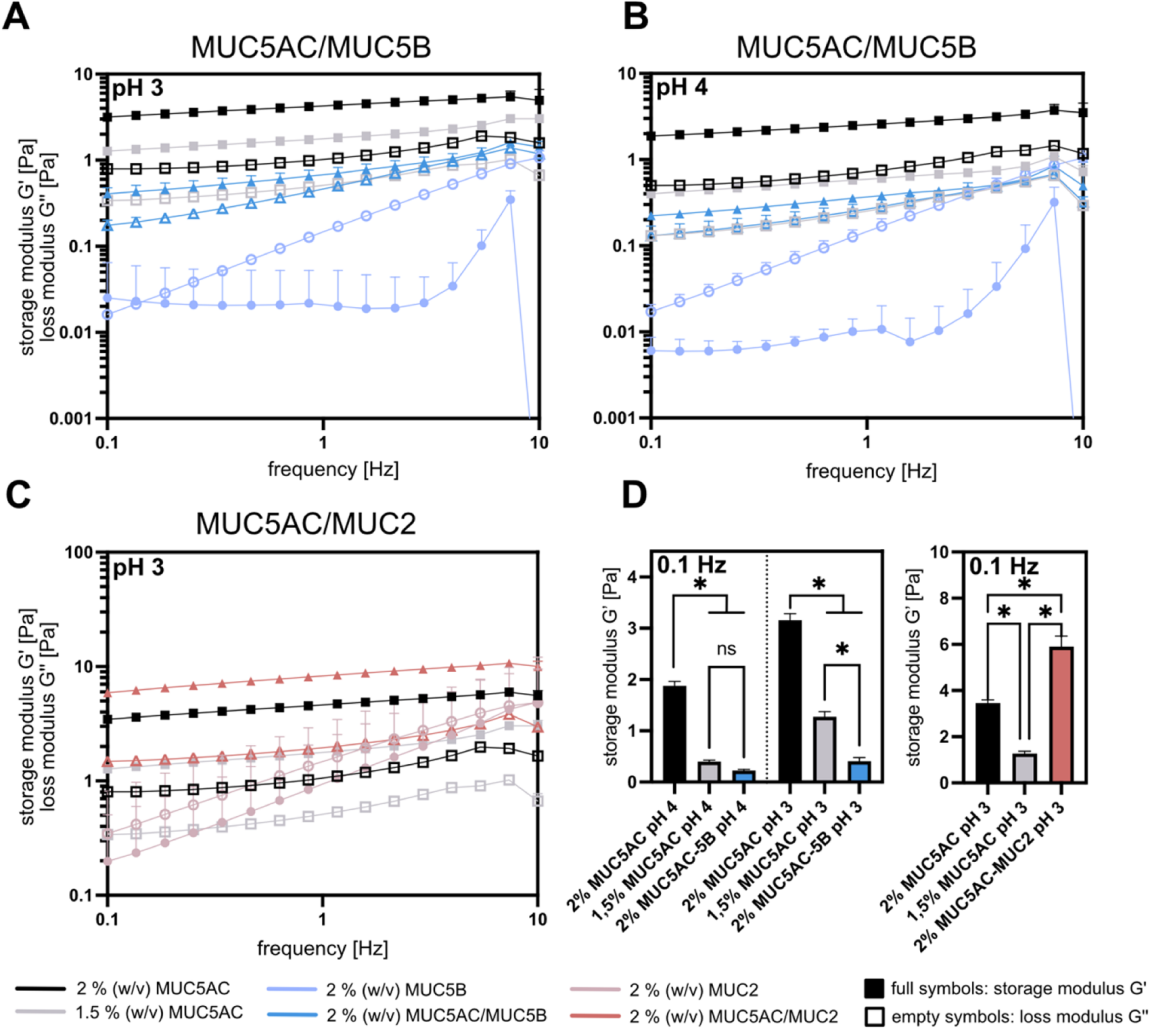
Viscoelastic properties of pure and mixed mucin systems. The macrorheological properties (as represented by the viscoelastic moduli *G*′ and *G*″) of mixtures of MUC5AC with MUC5B at pH 3 (A) and pH 4 (B), and with MUC2 at pH 3 (C) are compared to those of the respective pure mucin samples. (D) Direct comparison of the storage modulus *G*′(0.1 Hz) of selected samples from (A–C). Error bars indicate the standard deviation; if no error bars are visible, they are in the size range of the symbols. Asterisks indicate statistically significant differences, whereas “ns” indicates statistically nonsignificant differences based on a *p*-value of *p* = 0.05 (*n* = 3). Statistical significance was assessed using a two-sample *t* test; Shapiro–Wilk test and Levene’s test were conducted as pretests to assess the normal distribution and homoscedasticity of the data sets, respectively.

As the integrity of the gastric mucosal barrier critically depends on the ability of the mucins to form a stable hydrogel (in the stomach, this occurs under acid conditions), we conduct macrorheological measurements with pure and mixed mucin samples at a fixed overall mucin concentration of 2% (w/v) (this is within the physiological range of the mucin concentrations found in gastric mucus^32^). As indicated by the clear dominance of the storage modulus *G*′ (representing the elastic part of the material response) in the frequency spectra shown in Figure 2A,B, a pure MUC5AC sample forms a viscoelastic gel at both pH levels, pH 4 and pH 3. However, we observe a softening of the mucin gel when MUC5B is added to the MUC5AC host system: Even though the mixed system comprising MUC5AC and MUC5B sill forms a viscoelastic gel, the addition of MUC5B causes a significant reduction of the storage modulus by approximately 1 order of magnitude (see Figure 2D).

Interestingly, this weakening of the elastic properties of a MUC5AC gel induced by the presence of MUC5B is even more pronounced than it would be expected for a mucin gel reconstituted at a lower MUC5AC concentration (thus mimicking a scenario where the addition of MUC5B to the mixture were to simply reduce the MUC5AC concentration). Hence, it seems that MUC5B—which cannot form a viscoelastic gel at acidic pH itself (please note that the storage modulus measured for MUC5B solutions at pH 3 and pH 4 shown in Figure 2A,B is close to the lower measuring limit of the measuring device and should, therefore, be interpreted carefully)—does not only fail to contribute to the acidic gelation of the MUC5AC network, but actively interferes with the gel formation of the MUC5AC oligomers. Surprisingly, these observations are in marked contrast to results obtained for MUC2 contaminations of the MUC5AC host system. Here, even though MUC2 alone does not intrinsically form a viscoelastic gel at the conditions tested here either, MUC2 contaminations cause a significant stiffening of the mucin gel at pH 3 (see Figure 2C,D). This suggests that, different from MUC5B, MUC2 can efficiently substitute MUC5AC molecules in the hydrogel and may be well integrated into the MUC5AC network architecture.

As mentioned above, the mucus layer lining the gastric epithelium not only provides mechanical protection, but it also regulates the (diffusive) entry of substances from the stomach lumen into the tissue. Here, especially larger particles with a size above approximately 0.5 μm (thus exceeding the pore size of the dense mucin network) are prevented from traversing the mucosal barrier.^33^ Additionally, also the passage of particles/molecules smaller than the pore size of the mucin network can be selectively suppressed by the mucin network. This is largely achieved by Coulomb interactions: cationic particles can bind well to anionic moieties in the glycosylated region of the mucin macromolecules. In addition, small anionic and hydrophobic particles/molecules can be immobilized by mucins as well, and this is predominantly achieved by the hydrophobic and (locally) cationic terminal regions of the mucin polypeptide chain.^34,35^

**Figure 3:**
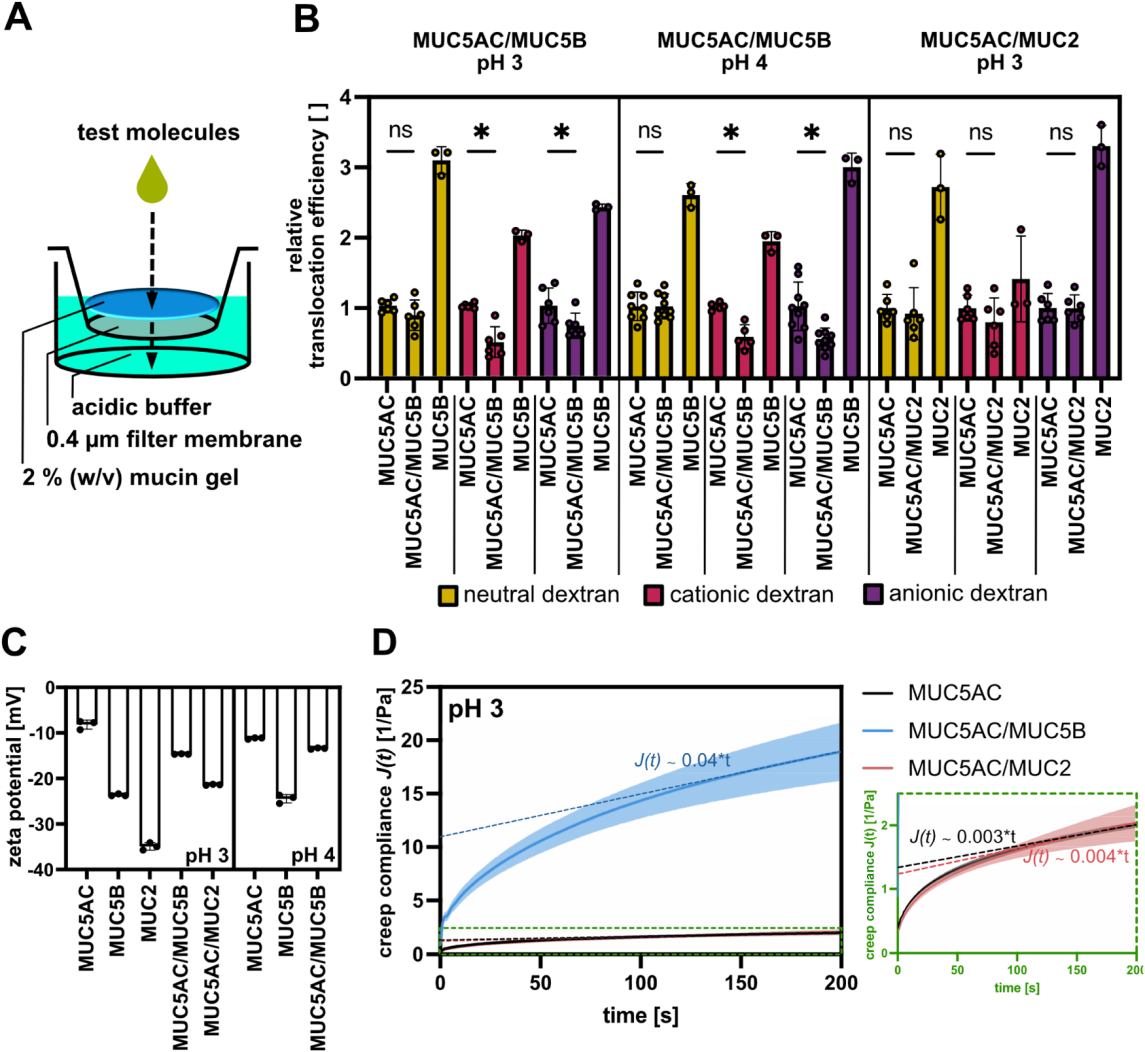
Barrier properties of pure and mixed mucin systems. A translocation assay (A) is employed to test the barrier properties of reconstituted mucin systems toward differently charged test molecules (uncharged/cationic/anionic dextrans). (B) The relative translocation efficiency of different reconstituted mucin systems is compared to the translocation efficiency of pure MUC5AC gels. The ζ-potentials of the different mucin variants are shown in (C). (D) Creep behavior of the different reconstituted mucin gels. Error bars in (B, C) as well as shaded areas in (D) indicate the standard deviation (*n* ≥ 3); Asterisks in (B) indicate statistically significant differences, whereas “ns” indicates statistically nonsignificant differences based on a *p*-value of *p* = 0.05; statistical tests were performed on a minimum sample size of (*n* ≥ 5). Statistical significance was assessed using a two-sample *t* test (with Welch’s correction for heteroscedastic data sets); Shapiro–Wilk test and Levene’s test were conducted as pretests to assess the normal distribution and homoscedasticity of the data sets, respectively.

To test the influence of atypical mucin species on the barrier properties of a MUC5AC host system, we investigate the translocation efficiency of a set of test molecules across an acidic mucin gel. For that purpose, we place a highly concentrated droplet of an acidic buffer solution containing differently charged dextrans on top of a thin mucin gel layer and compare the translocation efficiency of these molecules for MUC5AC gels with and without MUC5B/MUC2 contaminations (see Figure 3A). Owing to their small molecular weight of approximately 4 kDa, these dextrans are much smaller than the mesh size of the mucin network. Consequently, the efficiency of their diffusion driven translocation through a mucin gel is mainly influenced by binding interactions with the mucin molecules rather than by steric hindrance effects imposed by the mucin chains. Figure 3B shows the translocation efficiency of different dextran variants across pure and mixed mucin gels. For the experiments conducted here, the translocation efficiency indicates the amount of test molecules that have passed the respective mucin gel in comparison to the result obtained for a pure MUC5AC gel; in other words, translocation efficiencies >1/<1 indicate an increased/reduced translocation of test molecules through the respective mucin gel compared to the pure MUC5AC mucin gel and thus a weakening/strengthening of the mucin barrier brought about by the mucin contamination.

When comparing pure mucin systems reconstituted from MUC5AC, MUC5B, or MUC2 alone, we find that MUC5AC is much more effective in blocking the passage of any of the tested dextran variants than MUC5B or MUC2. The intrinsically weak barrier properties of MUC5B and MUC2 can, at least partially, be explained by their inability to form a viscoelastic gel at the tested pH levels (see Figure 2A–C). When we study mixed mucin systems, we make a surprising observation: even though all dextrans can easily pass a pure MUC5B barrier, the translocation of both cationic and anionic dextrans is significantly reduced in a mixed MUC5AC/MUC5B gel compared to a pure MUC5AC gel. At the same time, the translocation of uncharged dextrans remains virtually unaffected by the presence of a MUC5B contamination. Considering their small molecular weight of ∼4 kDa, the dextrans should easily be able to pass the polymer meshes of the mucin network. Hence, the diffusive motion of the dextrans within the mucin gel should not be hindered by size exclusion effects. Together with the observed charge dependent translocation behavior, this suggests that MUC5B modulates the barrier properties of MUC5AC gels toward small molecules by means of Coulomb interactions. And indeed, MUC5B has a ζ-potential that is more strongly negative than that of MUC5AC (see Figure 3C); therefore, the presence of MUC5B seems to add negative charges to the MUC5AC host system. These additional negative charges can rationalize to the increased immobilization of the cationic dextran variant but fall short of explaining the improved retention of anionic dextrans in the presence of MUC5B. This is curious, because we expected weaker electrostatic binding of the anionic dextrans to the strongly negatively charged MUC5B molecules than to the moderately anionic MUC5AC molecules.

Furthermore, when we add MUC2 to an MUC5AC host system, we do not observe a significant alteration in the barrier properties of the mixed gel compared to those of the pure MUC5AC host system—and this statement holds true for any of the employed test molecules. This result is surprising, since ζ-potential measurements suggest that MUC2 should add even more negative charges to the MUC5AC host system than MUC5B (see Figure 3C). Hence, there must be a more complex explanation that accounts for both, the altered barrier properties of MUC5AC in the presence of MUC5B contaminations and the absence of such alternations in the presence of MUC2 contaminations.

At this point, it is important to recall that the rheology data described above suggested that MUC5B interferes with the network architecture of the MUC5AC gel, whereas MUC2 appears to effectively substitute MUC5AC oligomers in the network architecture. We hypothesize, that there might be a relation between the viscoelastic properties of mixed mucin systems and their barrier properties toward charged molecules of both algebraic signs. One possible explanation for the results obtained with the translocation assay might be that the highly concentrated dextran solution added on top of the mucin gel layer locally exceeds the filtering capacity of the mucin network. As shown by Marczynski et al.,^34^ the ability of mucins to immobilize charged molecules is based on a finite number of oppositely charged binding sites that are either located in the glycosylated region of the mucin molecule (for binding of cationic molecules) and in the terminal region of the mucin molecule (for binding of anionic molecules). Overall, the number of cationic moieties in mucins is much lower than the number of anionic ones, and acidic pH levels are required for them to be present at all (so the side chains of the amino acids leucine, arginine, and histidine are protonated). However, once these binding sites for anionic molecules are occupied, the (local) electrostatic filtering effect is lost, and subsequent small molecules can pass the mucin gel.^20^

Such local overloading of the mucin filter capacity can, in principle, occur in any of the pure MUC5AC or mixed MUC5AC/MUC5B and MUC5AC/MUC2 gels studied here. The altered viscoelastic properties of the MUC5AC/MUC5B gel, however, might result from an increased chain mobility within the MUC5AC/MUC5B gel, and such an increased chain mobility of mucins could give rise to a gradual renewal of the local filtering capacity of the network over time (as “fresh” mucin chains enter areas of high dextran loading by diffusion/reptation and thus can trap molecules again). This may explain why both anionic and cationic molecules are more efficiently trapped in the mechanically softer MUC5AC gel containing also MUC5B. Hence, in a next step, we conduct creep tests to assess the polymer chain mobility in the different mucin gels.

When exposed to constant mechanical load, *i.e.*, a stress pulse, we expect to see an instantaneous jump in the creep compliance *J*(*t*), and this jump corresponds to the elastic part of the viscoelastic properties observed for all MUC5AC/mucin mixtures (as represented by the storage modulus *G*′ in the frequency spectra shown in Figure 2A,C). In contrast, at longer time scales, we expect to see creep of the different materials as typically represented by a linear regime in *J*(*t*). This creep originates from the viscous part of the viscoelastic properties and is linked to the reptation process of the polymer chains. In more detail, the polymer chain mobility should be related to the slope of this creep phase, which can be quantified by fitting a linear function to this section of the creep compliance *J*(*t*).

In line with our expectation, all tested mucin hydrogels show an elastic material response as characterized by the instantaneous deformation at *t*_0_; in full agreement with the frequency spectra discussed above, this instantaneous deformation jump is more pronounced for the softer MUC5AC/MUC5B gel than for the stiffer MUC5AC and MUC5AC/MUC2 gels. This elastic deformation is followed by an almost linear creep regime. Such creep is possible for the elastically dominated mucin networks since the elastic properties of the mucin gels studied here are predominantly established by hydrophobic interactions between the mucin termini,^15^ which can be considered to be transient cross-links that allow for a (local) reptation of polymer chains.^36,37^ Interestingly, the slope of the linear section of the creep compliance of MUC5AC/MUC2 networks is virtually identical to the slope obtained for a pure MUC5AC gel. In contrast, the corresponding slope of the creep compliance is approximately 1 order of magnitude higher for MUC5AC/MUC5B samples (see Figure 3D), thus indicating much stronger creep behavior. This finding supports our notion that the mobility of mucin chains within the MUC5AC/MUC5B gel is considerably higher than in pure MUC5AC gels or in MUC5AC/MUC2 gels.

Taken together, the results discussed so far suggest that MUC5B and MUC2 interact with MUC5AC in different ways, and that this gives rise to differences in the microarchitecture, viscoelasticity, and barrier properties of mixed MUC5AC/MUC5B and MUC5AC/MUC2 networks. But why is the interaction between MUC5B and MUC5AC so different compared to the interaction between MUC5AC and MUC2? To answer this question on a molecular level, we use AlphaFold 3^24^ to make structural predictions of the interactions between different mucin subtypes. Even though AlphaFold may not analytically solve the structure of protein domains but uses machine learning to predict the most likely structure based on known protein structures, it has been widely recognized for its accurate prediction results. Still, these predictions models are typically not capable of predicting the exact folding structure of complex proteins or protein–protein interactions. Hence, any prediction results need to be critically compared to experimental findings reported in literature.

Mucin-mucin interactions are usually established via their nonglycosylated, cysteine-rich terminal regions, where C-termini mediated dimers assemble into higher order oligomers through the N-terminal regions of the mucin molecules.^16,38−40^ C-terminal dimerization of mucins usually takes place before mucin secretion.^38^ Since we do not use any reducing agents to open existing disulfide bonds between the MUC5AC oligomers during the purification process, we assume that, in our mixed mucin system, the predominant mucin–mucin interactions are mediated by the N-termini, *i.e*., the assembly of preformed mucin dimers into oligomers. Accordingly, we employ AlphaFold to predict the structure of complexes formed by two or more mucin N-termini.

**Figure 4:**
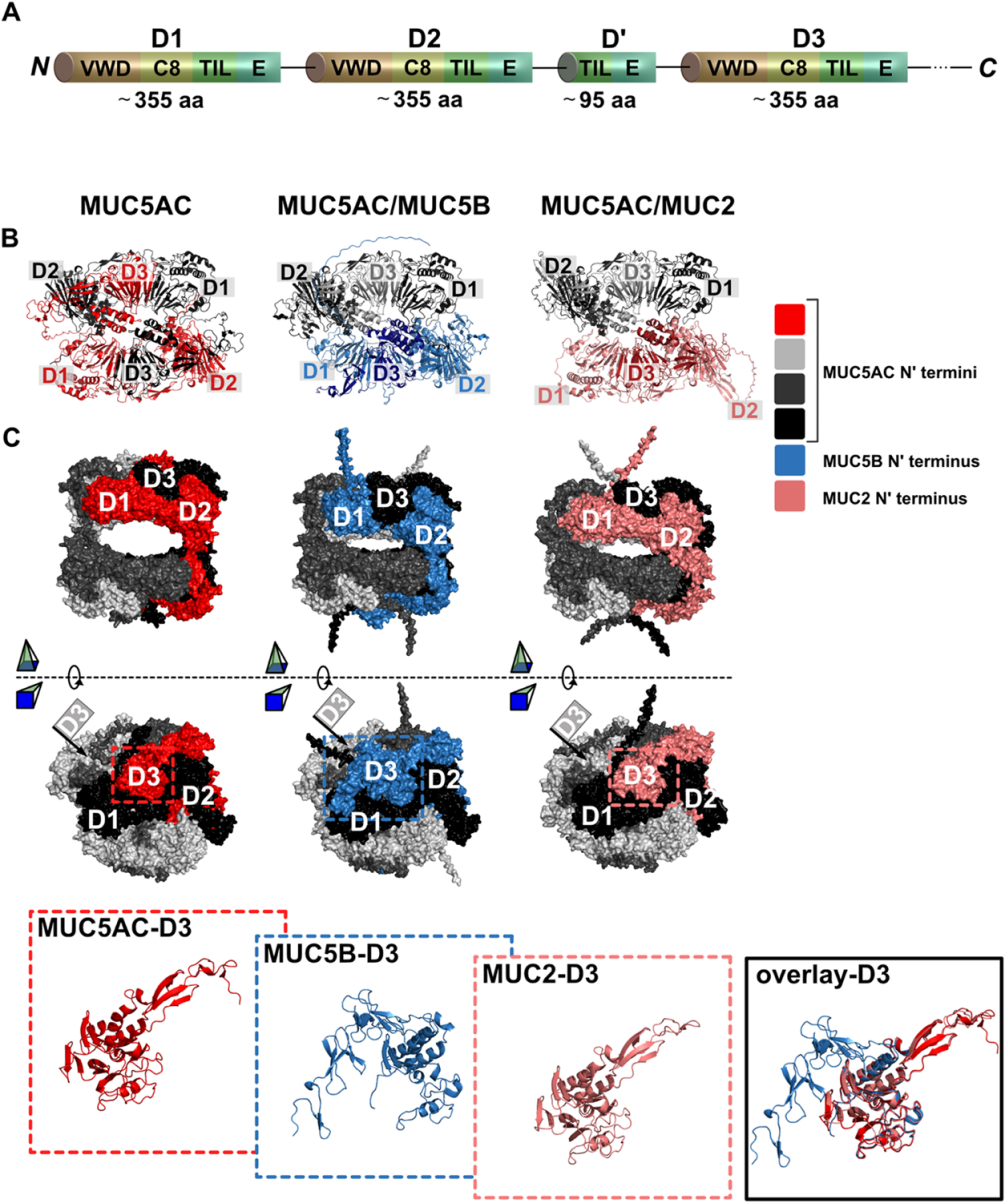
Structure of mucin termini and terminal mucin–mucin interaction. General building blocks of a mucin N-terminus (A) and predicted structure of N-terminal complexes comprising two (B) and four (C) N-termini of different mucin subtypes.

Independent from the mucin subtype, the mucin N-terminus comprises several subunits (D1–D3) as schematically illustrated in Figure 4A. Each of these subunits comprises several subdomains such as von Willebrand-factor like domains (VWD) or trypsin inhibitor like (TIL) domains;^41^ the structure of similar domains is known from other proteins and thus should allow AlphaFold to make reasonable predictions here. Also, all AlphaFold predictions analyzed in this study show consistent results (as at least four out of five possible structure predictions provided by AlphaFold for a given scenario are highly similar, see Supporting Information, section 6), which indicates that the predicted structures should be reasonably trustworthy.

As shown in Figure 4B, the N-terminal interaction of two MUC5AC molecules is driven by an interlacing of the proteins at the binding interface: here, the D3 domain of the first mucin’s terminus is situated between the D1 and D2 domains of the second mucin’s terminus. This result is in accordance with models of the N-terminal mucin–mucin interaction reported in the literature.^41,42^ For instance, Javitt et al.^42^ report a similar assembly based on cryo-electron microscopic images of recombinantly expressed MUC2 N-termini. This provides additional confidence in the accuracy of AlphaFold’s predictions made here. In a next step we analyze heterologous N-terminal parings between MUC5AC and one of the other two mucin variants. Interestingly, even though the amino acid sequence of MUC5AC clearly differs from the sequence of MUC5B (67% identity with MUC5AC) and MUC2 (50% identity with MUC5AC), the predicted structures of the heterologous pairs share a very high level of geometric similarity (for a detailed comparison of the amino acid sequences, the reader is referred to the Supporting Information, section 7). However, despite this high geometric similarity, we find an important difference: now, the D3 domain of one mucin terminus is not anchored between the D1 and D2 domains of the opposing mucin terminus but between the D1 and D2 domains of its own terminus. This could indicate that homologous mucin–mucin interactions might be preferred and more stable than heterologous mucin–mucin interactions.

In a second step, we simulate the interactions of several mucin termini (as required for the formation of a three-dimensional mucin network) focusing on tetramers. This is motivated by findings from the literature,^16^ where it was reported that MUC5AC intrinsically forms N-terminal mediated higher order multimers resulting in a highly branched network architecture. Similarly, MUC2 has been reported to assemble into trimers,^43^ which enables the formation of a branched network architecture. MUC5B, however, has been found to mainly organize into linear chains with a low degree of branching.^16,44^ The predictions made by AlphaFold indicate that both MUC5B and MUC2 may form tetramers with three MUC5AC termini as illustrated in Figure 4C. Thus, we hypothesize that, when integrated into a MUC5AC host system, the tendency of MUC5B to form linear, nonbranched chains may act as a chain-breaker in the MUC5AC network, thereby reducing the overall degree of branching. Such a reduced network complexity may adversely affect the network stability and yield a softer network with increased polymer chain mobility compared to pure MUC5AC or MUC5AC/MUC2 networks—which would explain our observations described above.

Furthermore, a closer evaluation of the relevant D3 domains in the tetrameric complex reveals a high structural similarity between the D3 domains of MUC5AC and MUC2 but strong structural deviations between the D3 domains of MUC5AC and MUC5B (see the overlay structures in Figure 4C). As a result, the D3 domain of MUC5B can be expected to not be well integrated to the tetrameric MUC5AC host complex. Fass et al.^41^ suggested that higher order N-terminal complexes are, *inter alia*, stabilized via interactions between neighboring D3 domains, which requires a proper alignment of these domains to enable disulfide bond formation. Hence, a weak integration of the MUC5B-D3 domain may reduce the stability of the MUC5AC/MUC5B complex and contribute to the generation of softer MUC5AC networks in the presence of MUC5B contaminations.

Our *in silico* analysis of the mucin–mucin interactions indicates that the molecular details of how foreign mucins are integrated into a stomach mucin host system are pivotal for the physical properties of the resulting mucin network. Here, we address N-terminal interactions between the mucins’ protein backbones. However, other interaction mechanisms, such as interactions between the O-glycosylated core regions of different mucin subtypes, could also be relevant in this context. These interactions are often mediated by additional peptides such as Trefoil factor (TFF) peptides or Galectin-2 (Gal), which are usually present in the gastrointestinal tract; changes in their expression profile are also associated with *H. pylori* infections and gastric cancer.^45,46^ Hence, these interactions could be interesting targets for future studies to experimentally investigate the range of molecular interaction mechanisms between different mucin subtypes. Particularly, small-angle X-ray scattering (SAXS) and fluorescence spectroscopy may help to further elucidate the structural organization of different mucin subtypes. Additionally, isothermal titration calorimetry or microscale thermophoresis could be valuable tools to compare the interaction affinity of homologous and heterologous mucin molecules.

## Conclusions

In this study, we analyze the interplay of the stomach mucin MUC5AC with two other major gel forming mucins, MUC5B and MUC2, and investigate physical properties of mixed mucin networks, in which MUC5AC is the main component. Physiologically, changes in the mucin composition of stomach mucus are associated with pathological conditions and can, *e.g*., be caused by bacterial infections with *H. pylori*.^10^ The high motility of *H. pylori* in stomach mucus enables the colonization of the deep mucosal layer close to the gastric epithelium.^47^ Our results suggest that changes in the mucin composition of gastric mucus might also induce undesired alterations in the mechanical and barrier properties there. For instance, a softening of gastric mucus in the presence of MUC5B as observed here for our model mucus system could facilitate the colonization of the mucosa by *H. pylori*. At the same time, increased barrier properties of MUC5B contaminated gastric mucus toward small, charged molecules could constitute a challenge for the therapeutic treatment of such bacterial infections. Moreover, *H. pylori* infections come with the risk of follow-up diseases such as chronic gastritis and gastric cancer,^48^ and gastric cancer is often characterized by the atypical *de novo* expression of MUC5B or MUC2.^11,12^ Our results motivate that the presence of those two mucins in gastric mucus may have very different consequences that need to be considered for the treatment of mucus-associated diseases.

## Supporting Information Available

Amino acid sequences of MUC5AC, MUC5B, and MUC2 used for the structure predictions; SDS PAGE with Coomassie staining for purified and repurified mucins; hydrodynamic size measurements of pure and mixed mucin solutions; turbidity of pure and mixed mucin samples over time; turbidity of MUC5AC/MUC5B samples at different mixing ratios; overlay images of the structure prediction results; alignment of the N-terminal amino acid sequences of MUC5B and MUC2 with the N-terminal amino acid sequence of MUC5AC (PDF)
