# Supplementary material for: Foreign Mucins Alter the Properties of Reconstituted Gastric Mucus
Source: Biomacromolecules. 2025 Feb 28;26(4):2293–303. doi: 10.1021/acs.biomac.4c01629 (PMC12004450; doi:10.1021/acs.biomac.4c01629)
Supplement: Supplementary file 1 — bm4c01629_si_001.pdf [file bm4c01629_si_001.pdf]

Supporting Information for

Foreign Mucins Alter the Properties of Reconstituted Gastric Mucus

Fabio Henkel[1,2] and Oliver Lieleg[1,2,*]

[1] School of Engineering and Design, Department of Materials Engineering,
Technical University of Munich, Boltzmannstraße 15, 85748 Garching, Germany

[2] Center for Protein Assemblies and Munich Institute of Biomedical Engineering,
Technical University of Munich, Ernst-Otto-Fischer Str. 8, 85748 Garching, Germany

*corresponding author:

Prof. Dr. Oliver Lieleg

Center for Protein Assemblies and Munich Institute of Biomedical Engineering,

Technical University of Munich,

Ernst-Otto-Fischer Str. 8, 85748 Garching, Germany

e-mail: oliver.lieleg@tum.de,

phone: +49 89 289 10952, fax: + 49 89 289 10801

Keywords: heterologous, ectopic, site unspecific, gastric cancer, lung mucus, intestinal mucus

ORCIDs: **FH** (0000-0001-7433-4802); **OL** (0000-0002-6874-7456)

1 Amino acid sequences:

The amino acid sequences of the three different mucin variants were obtained from the uniport database as listed below. In each sequence, the first 1100 amino acids marked in red correspond to the N-terminus used for the sequence alignment and the structure predictions conducted in this study. The underlined sections correspond to the D3 domains.

MUC5AC:

Uniport entry: A0A8W4FM62_PIG [1]

Length: 4915 amino acids

Sequence:

GGCVSPALFPVSPGDLLRKVTVHPPLRTTPVVRALNAAHGGQVCSTWGDFHYKTFDGDVFRFPGLCNYV

FSAHCGAAYEDFNIQLRRGPAPNATAPSRVTMKLDGMVVELTKSSVLVNGRTIQLPFSQSGVLVEQSSSY

VKVVARLGLVFLWNQDDSLLLELDAKYANKTCGLCGDFNGIPVFNEFLSHGKLSPIEFGNLQKMDGPME

QCQDPVPESPVNCSTSSGICKEMLSELFPGCAALVDASSYLNACQHDLCRCQQANLTSCLCHTLAEYSRQ

CAHAGGQPLDWRGPHLCPQTCPQNTEYRECGSPCADTCSNLEHSQLCEDHCVAGCFCPKGMVLDDVG

HTGCISVSECSCVYNGVTYAPGTGYSTDCSSCTCSGGRWSCQEVPCPGTCSVLGGAHFSTFDEREYTVHG

DCSYVLAKPCNSSAFTVLAELRRCGLTDSETCLKSLTLSLAGGQTVIVVKASGEVFVNQIYTQLPVSTANVM

LFRPSTFFIIAQTQLGLQLDVQLVPVMQVFVRLAPQLRGHTCGLCGNFNSIQADDFRTISGLVEGTAAAFA

NTWKTQAACPNIKNNFEDPCSLSVENEKYAQHWCSRLTDTHGPFARCHAAVNPSTYYSNCMFDTCNCE

KSEDCMCAALSSYVRACAARGVLLSGWRDGVCTTPTATCPKSLTYRYHISTCQATCRARSDEGDATCSVS

FVPVDGCTCSNDTFLDDTGKCVPATSCPCYYRGSVVPNGESLHDGGAVCTCTQGTLACIGGHDPTPVCV

PPMVYFDCRNATPGATGAGCQKSCHTLDMDCYSSQ<u>CVPGCVCPSGLVASGEGGCIPASDCPCVHNEAS</u>

<u>YPAGQTIRVGCNTCTCKNRTWQCTDQPCLATCAVYGDGHYLTFDGQRYSFSGDCEYTLVQDHCSGNGS</u>

AQDGFRVITENIPCGTTGTTCSKAIKLFLGSDELKLSDGKVEVIETDPGQPPPFAIRQMGIYLVVDTDAGLV

LLWDKKTSIFLTLSPEFKGRVCGLCGNFDGNALNDFTTRSQSVVGDVLEFGNSWKFSPSCPDARAPKDPC

TANPYRKSWAQKQCSIINSATFSACRAHVEPARYYEACVSDACACDTGGDCECFCTAVAAYAQACHEVG

VCVSWRTPDICPLFCDYYNPQGQCEWHYLPCGAPCMRTCRNPRGQCLHDIQGLEGCYPKCPPEAPIFDE

DQMHCVTSCPTPPPPAPCRVQGKTYRPGSTVPSDENCHSCVCTVSGVQCTYDPDACVCTYDGRRFRPG

EVIYHTSDGTGGCISARCGANGTIDRGVSACSPATPTPQTTFVFSTSPLVVSSTLPPSTRPSPATSSTHTPSR

ASLTTPGTPPGPTPHCGEECLWSPWLDVSRPGLGIDSGDFDTLENLRAHGYRVCRAPSAVECQAEDAPG

VPLRALGQRVECSPTVGLICYNRDQPSGHCDNYQIRILCCSPRACPPGSTATSPPPAFNTTETGPTPGTSGI

TPGPGTPTPISGTSTPGPGSPTPAPGSPTPAPGTSTSGPGTPTPGPGTPTPAPGTSTSGPGTPTPAPGTPT

PVPGSPTPGPGTPTPAPGTSTSGPGTPTPAPGTSTSGTPTPAPGTSTSGPGTPTPGPGTPTPVPGSPTPGP

GTPTPAPGTSTSGPGTPTPAPGTSTSGPGTPTPGPGTPTPAPGSPTPTPSISTSGPGTSTSGPGTPTPAAG

ASTSGPGSLTPVPGTSTTVKTTIWTPSTTAVSTTTSVVPSKPTSHEPSPVTCLQESCTWTKWIDGSYPGPD

RNSGDFDTFQNLRAKGYRFCAKPVNVECRAESFPDTPLQALGQDVICDKTVGLVCLNKDQLPPICYNYEIR

ILCCEMVDTCLRSTTEPFTPESTRQSSSAWTPGVVSPSTQHSTATSGHTPTASSITSSWHGPTPPSLTPCRP

QCSWSKWFDVDFPSPGPHGGDFETYSNILRSGEKICRQPEYISDLQCRAQNHPEVSIQKLGQVVECRPEV

GLVCRNQDQGGKFRICLNYEVRVLCCEPKKDCPVSPITLPTTTSVRVTSPPETSSHGATSSTTSVQPSSSSSP

PISSTTSVQSSSSSSALLQPPVQPLCSPAAPAHLQYPAPSLCRQAAPAQLQPPVQPLCRQAAPAQRQYPA

PPRCSQAAPAHHQPPVPPLCSQAAPAQLQYPVPPLCSQAAQALLQPPVQPLCRQAAPAHLQYPAPSLCR

QAAPAQCPPPAPPLCSQAAPAQLQPPVQPLCRQAAPAQRPPPVQPRPLFLGPPLPHAAPRRVGPSAPG

PSGSTWTSRPPGPTEETLRPTAISSEAEKRSAGNLSTSATCSAEPRTTPRSASRSWARWWSAGRSGPGVQ

EPGPGGKFRICLNYEVRVLCCEPKKDCPVSPITLPTTTSVRVTSPPETSSHGATSSTTSVQPSSSSSAPTTSAT

SVRSSSSSSAPIPSTTSVQPSSSSSPPISSTISVQPSSSSSVPTTSTTSVQPSSSSSVPTTSATSVRSSSFSSTAIP

STTSVQPSSSSSSPTTSTTSVQPRSSGSAPTTSATSVQSSSSSSPPISSTISVQPSSSSSVPTTSATSVQPSSSSS

PPISSTISVQTSSSSSVPTTSTTSVQPSSSSSAPTTSATSVHTSSSSSAPTTSATSTSVPGSTSSSRSPTPCQPQ

CSWSKWFDVDFPSPGPHGGDFETYSNILRSGEKICRQPEYISDLQCRAQNHPEVSIQKLGQVVECRPEVG

LVICLNYEVRVLCCEPKKDCPVSPITLPTTTSVRVTSPPETSSHGATSSTTSVSSSSAPTTSATSVQPSSSSSPP

IPSTISVQPSSSSSAPTTSATSVQPSSSSSPPISSTVSVQTSSSSSVPTTSTTSVQPSSSSSVPTTSATSVRSSSS

SSTPIPSTTSVQPSSSSSAPTTSATSVQPSSSSSTPIPSTTSVHSSSAPTTSATSTSVPGSTSSSRSPTPCQPQC

SWSKWFDVDFPSPGPHGGDFETYSNILRSGEKICRQPEYISDLQCRAQNHPEVSIQKLGQVVECRPEVGL

VCRNQDQGASSGSASTMRCVCSAVSPRKTALSPSSSSSVPTTSTTSVQPSSSSSVPTTSATSVRSSSSSSTPI

PSTTSVQPSSSSSVPTTSATSVQTSSSSSTPIPSTTSVQPSSSSSAPTTSATSVQPSSSSSPPISSTISVQPSSSS

SSPTTSTTSVQPSSSGSAPTTSATSVQPSSSSSPPISSTISVQPSSSSSAPTTSATSVQSSSSSSAPTTSATSVQ

PSSSGSAPTTSATSVQSSSSSSPPISSTISVQTSSSSSVPSTSTTSVQPSSSGSAPTTSATSVHTSSSSSTPIPST

TSVQPSSSGSVPTTSATSVQSSSSSSAPTTSATSVSATSTSVPGSTSSSRSPTPCQPQCSWSKWFDVDFPSP

GPHGGDFETYSNILRSGEKICRQPEYISDLQCRAQNHPEVSIQKLGQVVECRPEVGLVCRNQDQGGKFRI

CLNYEVRVLCCEPKKDCPVSPITLPTTTSVRVTSPPETSSHGATSSTTSVQPSSSSSAPTTSATSVQPSSSSSA

PTTSATSVQPSSSGSAPTTSATSVQSSSSSSPPISSTISVQTSSSSSAQTTSTTSVQPSSSGSAPTTSATSVHTS

SSSSTPIPSTTSVQPSSSSSAPTTSATSVQSSSSSSPPISSTTSVQPSSSSSPPISSTTSVQPSSSSSPPISSTTSV

QPSSSSSAPTTSATSVQSSSSSSAPISSTTSVQPSSSGSVSTTSATSVQTSSSSSPPISSTISVQPSSSSSVPTTS

ATSVQPSSSSSAPTTSATSVQTSSSSSTPIPSTTSVQPSSSSSAPISSTISVQPSSSSSSPTTSTTSVQPSSSGSA

PTTSATSVQPSSSSSPPISSTISVQPSSSSSVPSTSTTSVQPSSSGSAPTTSATSVHTSSSSSTPIPSTTSVQPSS

SSSAPTTSATSVQSSSSSSAPTTSATSTSVPGSTSSSRSPTPCRPQCSWSKWFDVDFPSPGPHGGDFETYS

NILRSGEKICRQPEYISDLQCRAQNHPEVSIQKLGQVVECRPEVGLVCRNQDQGGKFRICLNYEVRVLCCE

PKKDCPVSPITLPTTTSVRVTSPPETSSHGATSSTTSVQPSSSSSPRLGVSCWPGSSHLLLPVPGSIIYEETDL

SGHCYYAVCSLACRVVRRTDLSCPTSRPPPASSTPRPGSSPSSHVPQHGCPNAVPPRMKGETWPMPNCT

EASCEGNGVISVRPRHCPKVQKPTCANGYPAVKVAKPEGVCSHLSATSSVCSGWGDPHYITFDGTYYTFL

DNCTYVLVQQIVPVYGHFRVLVDNYFCGAEDGLSCPQSIIVEYQQDRVVLTRRPVRGVMTNQIIFNNEVV

SPGFRKDGIVVSQVGIKMYVAIPEIGVQVMFSGLIFSVEVPFSKFANNTEGQCGTCTNDQKDECRLPGGA

VVASCSDMSSHWKVTLPGQPPCHAPPPRPTVVEPTTPPTSCPPSPICQVFAPCHAEIPPWPFFQGCVFD

HCHMPDTDVLCSGLELYAALCASLGVCIDWRGRTNHTCPFPCPADTVYQPCGPSNPPYCYMNNSANAL

ALPEAGSITEGCFCPQGTMRFSTGSEVCVPADCSWCLGPHGEPVEPGHTVSFDCQECSCDGHTRTVSCR

SQTCPLPPACQEPGLVPVPEALQSGQCCPQYSCACNTTRCPVPVECPEGSHLVLTYEEGACCPSYSCNWT

SCSVNGTLYQPGAVVSSTLCETCRCEVPGGPESDTFAISCETQICSTYCPVGFEYQERQGQCCGFCKQVAC

VTNTSDSSVHLFYPGESWSDPGNRCVTHECEKHQEGLVVVTTRKACPPLTCAKDQAQLSKDGCCLFCPR

RTKAVSCAVYHQHQVLQQQSCRSAGPVRLTYCQGNCGDTASMYSPEANAVEHRCKCCQELQVALRNV

TLHCPDGSSRAFSYTEVEKCGCVGQRCDSHGDLSLSEEEAPQLSRDAGHGLWRTGAPQPRPLQ

MUC5B:

Uniprot entry: F2FB42_BOVIN [2]

Length: 6724 amino acids

Sequence:

MGALSGCPALLWALVALLSAGTADTQDGEQQSWTSQQPEMTARHLTFIPPITVFPTMSPLNAAHGGRV

CSTWGDFHYKTFDGDVFRFPGLCNYIFSAHCGSAYEDFNLQLRRGLLGSRPTITHIVLRSQGLVLEVSNGS

VLINGWREELPYSRAGLLVERSSTYVKINIRLMLTFMWNGEDSALLELDPKYANQTCGLCGDFNGLRAVS

EFYAHNTRLSPLQFGNLQKLDGPTEQCQDPLPSPAADNCTDEGGSHISTYDEKLYDVHGDCSYVLTKVCA

DSALTVLAELRKCGLTDNENCLKTVTLSLNGGDTTVQIQANGGVFVNSIYSQLPMSVADVTVFRPSSFFIL

VQTGPWLQLQVQLVPLMQVFLRLDPAYRGQMCGLCGNFNQNQADDFRTVSGVVEATAAAFANTWK

TQAACPNVKNSFEDPCSLSVENENYAQHWCSLLTRPAGPFSPCHSVISPGPFHSNCLFDTCNCEKSEDCM

CAALSSYVQACAARGVLLSGWRDGVCTKYASSCPKTQSYAHVVDSCQPTCRSLSQPDVSCDVAFVPVDG

CVCPRGTFLDDAGTGVPAEACPCYLRGTVLAPGEVVHDNGVVCSCVSGRLSCLGATEQSTGCVAPMVFL

DCSNASADAPGAECVRSCHTLDVDCFSTHCVSGCVCPVGLLSDGSGGCVAEEDCPCMHNEAAYKPGEVI

KVDCNTCTCRGRRWECSDRPCLGTCVAYGDGHFLTFDGERYGFEGSCEYTLAQDYCVGSDTANGTFRIV

TENVPCGTTGVTCSKAIKIFLGSYELILHEGTHRVLQRGPGGDLPYRVRYMGIYLTVETHGGVVVSWDRKT
SVIIRLRHEYKGRVCGLCGNFDDNALNDFTTRSQSVASDVLEFGNSWKFSPSCPDALAPRDPCTTNPYRR
SWAQKQCSIINSATFSACRSQVDPTRYYEACVSDACACDSGGDCECFCTAVAAYAQACHEAGVCVSWR
TPDVCPLFCDYYNPHGQCEWHYQPCGAPCLKTCRNPSGLCLMDLPGLEGCYPKCPSSKPFFNEDQMEC
VAQCSGCYDGDGNYYDAGTRVPSTENCQSCDCTSSGLQCTHSPEACTCTYEGRTYAYGDVIYNTTDGLG
ACLIAICRDNGTIVRRAVECPGTLFKTPFTFTSTAAPPSTTGLVPTPSTVCVRKVCHWSDWYDGGHPEPG
MSGGDFETFENLRQRGYQVCLAPVDIECQAQLLPGIPLEELGQKVECSRDKGLTCFNSEQSPPLCLNYKLR
VLCCDYVPCGTSQPPSSQTTPTTGTTHPTMAVTTSQSPSTKLTTNTTPEGSSSVFPATTTCEPHCHWTEW
FDVDYPKYEEGGGDLETYEKIRGAGGAVCKQPQEIECEAENYPGLTPEQVGQQVHCDVRFGLVCRNDEQ
LGLFKMCYNYRMRVLCCEYNHCTLPTATTSPTATATASTATAGTATTAAVPTTTTATVPTATTSTATITVP
AATTSTATATVPTATTATVPTATVPTATTATVPTATVPTATTAAVPTATVPTATTATVPTATVPTATTAAV
PTATASTATAATATTATVPMTTSSIATATTATARTPTTSTPAPTTATTGATTSQGTTSCQPKCKWTEWFD
VDYPKYEEGGGDFETYEKIRGAGGAVCKQPQEIECEAENYPGLTPEQVGQRVHCDVHFGLVCRNDEQLG
LFKMCYNYRMRVLCCEYNHCTLPTATTSPTATATASTATAATATVPTATPATTTSATVPTATTATVPTATT
STATTTVPIATTTTATVPTENATTVTVSIATPSTAPGTTTTAPTATVPTATTATVPTATTATVPTATAMSAT
VPSATTAAVPTTTASIATATTVTAPTSTVPTATTSTVPTVTATTETVSTATASTATAATATTTGTATTATVPT
ATTATVPMATTSTATATVPTATTATVPTATTATVPTETTTTATVPTATTATTTTATVPRATTSTATATTATT
ATVPKATTATVPMAATSTAIVPRATTATATTTTVPTATTSTATAVTATTATVPTATTTTATISTAATSNATA
PTAAVVTATVPTAAISTGTVPTASAASSTTSTAVPTPTQTRTQTGSTVVTGSPASSATPVPALSTRLTTRVT
ALSPGQTGTHPTSPPGSTGSTSPGTAMSVLPTRKTSQGPTSVSATTQTSTARLPTETAVSTTYTPSTQHPE
TSTPAPTTATTGATTSQGMTSCQPKCKWTEWFDVDYPKYEEGGGDFETYEKIRGAGGAVCKQPQEIECE
AENYPGLTPEQVGQRVHCDVHFGLVCRNDEQLGLFKMCYNYRMRVLCCEYNHCTLPTATATTATVPTA
TMATTTTATAPTATMVTVPTATTSTATMPTATMATVPMATPSTSTATVPTTSTATTTVPIATTTTATVPT
ENATTVTVSIATPSTAPGTTTTAPTATVPTATTTTATVPTATTATVPTATATSATVPSATTATVPTTTASIAT

ATTVTAPTSTVPTATTSTATAVTATTATVPTATTATVPTATTATVSMATTTTTASVPTATTATVPTATTTTT
ATVRTPTTATVTMATATTATVPMATISTATATVPMATTAIATISTAATSNATAPTATVVTATVPTAAISTG
TVPTASAASSTTSTAVPTPTQTRTQTGSTVVTGSPASSATPVPALSTQLTTQVTASSSGHTGTHPTSPPGS
TGSTSPGTATSVLPTRKTSQGPTSVSATTQTSTARPPTETAVSTTRTPSTQHPETSTPAPTTATTGATTSQG
MTSCQPRCKWTEWFDVDYPKYEEGGGDFETYEKIRGAGGAVCKQPQEIECEAENYPGLTPEQVGQRVH
CDVHLGLVCRNDEQLGLFKMCYNYRMRVLCCEYNHCTLPTATTSPTATATASTATAGTATVPTATTATTT
TATVPTATTATVPTATPATTTSATVPTATTATIATVPTATTTTVPMATTATMPTATTATVPTATTVTTTTA
TVPTATTATVPTATTATVPTATTTTATVPTATTATVPTATSSTSTTTVPMATTYTATAGTATTAEVPMATT
TTAIVPTATTAAVPTATTTTATVRTAATSTATVPTATTATVPTATTATVPTATTTTATVPTATTATVPTATS
STASAATATTAEVPMATTTTAIVPTATTATVPTATTTIATVPTATTATTSTATVPTATTATVPTATTASVPM
ATTATVPSATTSTATISTAATSNATAPTAAVVTATVPTAAISTGTVPTASAASSTTSTAVPTPTQTRTQTGS
TVVTGSPASSATPVPALSTRLTTQVTASSPGQTGTHPTSPPGSTGSTSPGTAMSVLPTQKTSQGPTSVSAT
TQTSTAWPPTETALSTTRTPSTQHPETSTPAPTTATTGATTSQGTTSCQPKCKWTEWFDVDYPKYEEGG
GDFETYEKIRGAGGAVCKQPQEIECEAENYPGLTPEQVGQQVHCDIRLGLVCRNDEQLGLFKMCYNYRM
RVLCCEYNHCTLPTATTSPTATATASTATAATATVPTATPATTTSATVPTATTATIATVPTATTTTVPMATT
ATVPTATTATVPTATTTTATVPTATTATVPTATTATTTTATVPTATIATVPTATATTATVPTATTATTTTAT
VPTATTATVPMATSSTATTTVPMATTYTATAGTATTAEVPMATTTTAIVPTATTAAVPTATTTTATVPTA
TTTIATVPTATTATTSTATVPTATSATVPTATTASVPMATTATVPSATTSTATISIAATSNATAPTAAVVTAT
VPTAAISTGTVPTASAASSTTSTAVPTPTQTRTQTGSTVVTGSPASSATPVPALSTRLTTQVTASSPGHTGT
HPTSLPGSTGSTSPGTATSVLLTQKTSQGPTSVLATTQTSTARLPTEIALSTTYTRSTQQPETSTPAPTTATT
GATTSQGTTSCQPKCKWTEWFDVDFPTSGVMGGDIETYDNIRAAGGKMCQDPEKIECRAENYPEVSID
QIGQVLNCSLETGLVCRNEDQRGPFTMCFNYNIRVYCCDDVRHCPTTATPGLRSTSLPPGSSTATGPWPP
TSTTTWPPFSSSKVLPMTPKPTLTGAPSTVTVVTSHILESTSVTNLPPSPVMPTTRATTHTQFLPPTTSKGM
TQITELPTRPSETTTRPPVTTLSTGQATSHTGVGTSTSLSPPGPEPSTSSLGTTLGPTTTTTSMATLSSPKTHT

SGTTLVPTSPGTESTTCERRCAWTDWMDKSYPMPGAYGGDYETYANIQAAGGAICEQPLKLECRAEELP

DIPLPDLGQVVLCQLEVGLVCRNQDQSGQMCLNYQIRLLCCDKSYCPSTAITTSTPTSETGLSSTVTTGRVS

IPTSVTGPSSTVTTERVSTPMSLPGPSSMVTSGVVSTTTSVTGPSSKATTKRVSTRTSVTGPSSMVTTKRTS

TSTSVTGPSSTATTERVSTPTSVPGPTSTVTTERVSTPTSVPGPSSTVTTERVSTPTSVPGPSSTVITERVSTA

TSVTGPSSIATTERVYTPTSVPGSSSRVTSAAVSTSTSVTGPSSMVTTERTSTPTSVTGPSSTATTEKVSSPTS

VTGPSSTATTERTSTPTSVTGPSSTATTEKVSTPTSVTGPSSTATTERVSTPTSVPGSSSTVTTERTSTHTSVT

GPSSTVTMERTSTPTSVTGPSSTATTEKVSTPTSVTGLSSTVTTEGVSTPTSVTRPSSTATTERTSTPTSVTG

PSSTVTTEGVSTPTSVTGPSSTATTERTSTPTSVTGPSSTATTERVSTPTSVTGPSSTATTERTSTRTSVTGPS

STVTTEGVSTPTSVTGPSSTATTERTSTPTSVTGPSSMVTTKRTSTSTSVTGPSSTATTERVSTPTSVPGPTS

TVTTERVSTPTSVPGPSSTVTTERVSTPTSVPGPSSTVITERVSTATSVTGPSSIATTERVYTPTSVPGSSSRV

TSAAVSTSTSVTGPSSMVTTERTSTPTSVTGPSSTVTIEGVSTPTSVTGPSSTVTTERTSTPTSVTGPSSTATT

EKVSTPTSVTGPSSTATTERTSTPTSVTGPSSTVTTEGVSTPTSVTGPSSTATTERTSTPTSVTGPSSTATTER

VSTPTSVTGPSSTATTERTSTPTSVMGPSSMVTTERVSTPMSVPGPSSTVTTERVSTPTSVPEPSSTVTTER

FSTPTSVTGPSSTETTERTSTPTSVTGPSSTVTTEAVSTPTSVTGPSSTATTERTSTPTSVTGPSSIATTERVYT

PTSMPGPSSTVTSAAVSTSTSVTGPSSMVTTERTSTRTSVTGPSSTATTERVSTPTSVTGLSSMVTTERTST

PTSVPGPSSTATTERTSTPTSVTGPSSTATTERVSTPTSVPGSSSTATTERTSTHTSVTVPSSTVTMERTSTS

TSVTGPSSTVTTEKVSTPTSVTGPSSTVTTEGVSTPTSVTGPSSTATTERTSTPTSVTGPSSTVTTEGVSTPTS

VTGPSSTATTERTSTPTSVTGSSSTATTERVSTPTSVMGPSSTVTTERVSTPTSVPGPSSTVTTERVSTPTSV

PGPSSPVITERVSTPTSVTGPSSIATTERVYTPTSVPGSSSRVTSAAVSTSTSVTGPSSMVTTERTSTPTSCDR

TLLHSDHREGLHPHECARTHLHGDHREGVHPNKCARTLLHATTERVYTPTSMPGPSSTVTSAAVSTSTSV

TGPSSMVTTERTSTRTSVTGPSSTATTERVSTPTSCARILLYRDHGENLHPHQCHRTIFHGDHGEDLHPHQ

CDRSLLHSDDGEATTERVSTPTSVPGSSFTATTERTSTHTSVTGPSYTVTMEKCARILLYRNHGENLHPHQ

CHRTLFHATTERVSTPTSVPGSSSTVTTERTSTHTSVTGPSSTVTMERTSTPTSVTGPSSTATTEKVSTPTKT

SGSLMPSIVPTTSPGPTTPCLCHAFGKFFLPGDIVYNKTDGAGCPFLAICNQRCDLDRFQGACTTSSPPGT

SASVPPTTPLSDCDRTIPPRKVNESWFLEDCTVARCEGDNRVTLLGPRPMSSITCVNGHLPVKVQNQSHR

CDYHYECECVCSGWGDTHYETFDGTSYSFWDNCTYVLMREIQPRHGNLRILLHNRFCEATAHCPRALSIH

YQSVDIVLSTTSSAAGQEESLILLDQTRMRQSFSKNGVIVTLTGATGMRVDIPAVGVGITFNGRVFQARLS

YSRFSHNTEGQCGTCTNNRRDECRRPDGTTARTCRDMARSWLVSNSSVEGCGVPTGLPPTTSPLLPETS

TPAIHPSCPPEPLCELMLSSVFAGCHSLIPPGPYFNACVSDSCWPGRGRKVLCQSLEAYAELCRSRGVCPD

WRNATHGLCDLTCPSAKVYKSCGPVQPESCDSRSQSPLSVGLAEGCFCPDGHILFNSHRDICVPECPCVG

PDGLPKFPGERWVSNCQDCVCDNGTVSVQCTPVECLAPDQPQQCGRAGFVAVSRPLADNPCCMENLC

VCNASTCPQSPPACRLGEKLVRTQVEDDCCPTFSCEPLLCTFNGTFYGVGATIPGVTPCHTCTCLSMDSED

LTVRCEEEACNTTCLQGFEYSTVAGQCCGECVQTACLAPDGQLVQLNETWVNSLVDNCTEYHCQARDG

PPMLTPMPVVCPDGGQVHSNMTVLRHRGCVTAVKVSFCEGSCPKSVPGASHCLRTPKRLGAGYSMEA

QARQCSCSCCQETRTHQEVVTMQCPDGTAFQHTYTHVDECSCVPACTPPHHGNHECPGMPSMGTTA

PALFALGLVKPGPYRVTAQRRRRCAKNRTPLRSGPTAASVLLEAPRAQTPTGYLLQRTTD

MUC 2:

Uniprot entry: A0A8D1ICB6_PIG [3]

Length: 4222 amino acids

Sequence:

MGLPLARLVALCLALTWAGGAELQREGRTRNHGHNVCSTWGDFHYKTFDGDVFRFPGLCDYNFASDC

RDAYKEFAVHLRRGPGGSGGPSQVEYILLTVKDDTIYLTQQLVVVNGAMVSTPHYSPGLLIERSAIYTKVYS

RAGLALVWNREDSVMLELDSKFQNHTCGLCGDYNGLQTYSEFLSEGIPFSPLEFGNMQKINKPEEKCDD

PEEAQAKLSCSEHRAECERLLTDVAFEDCQGLVPLELYVQACVQDRCQCPQGTSCVCSTIAEFSRQCSHA

GGRPGNWRTATLCPKSCPGNMVYLESSSPCVDTCSHLEVSSLCEEHRMDGCFCPEGTVYDDIAGRGCIP

VSQCHCKLHGHQYAPGQQVTNNCEQCVCNAGRWVCQDLQCPGACALEGGSHITTFDGRKYTFHGDC

YYVLTKGTHNDSYAILGELTPCGSTDKQTCLKTVVLLADNKKNVVLFKSDGSVLLNELQVNLPHVTASFSIF

QPSSHHLLVDTAFGLRLQVQLAPMMQLFLTLDQAAQGRVQGLCGNFNGLEGDDFKTAGGLVEATGAG
FANTWKAQSSCHDKVDWLDDPCSLNIESANYAEHWCSLLKKTGTPFGRCHSAVDPAEYYKRCKYDTCN
CQNTEDCLCAALSSYARACAAKGIMLWGWREHVCNKDVGSCPQSQIFRYNLTTCQQTCRSLSEADAHC
LEGFAPVDGCGCPDQTFLDEKGRCVPLAKCSCYHRGLYLEAGEVVLRQEERCVCRSGRLHCVPVKLLGQS
CEAPKIHVDCNNLTALAIRNPRPVSCQTLAAGYYQTECVSGCVCPEGLIDDGRGGCVVEEACPCVHNEDL
YSPGDRIRVDCNTCTCRKGRWACTQSVCHGTCAIYGSGHYITFDGKYYDFDGHCSYVAVQDYCGQKSPQ
GSFSIITENVPCGTTGVTCSKAIKIFLGRTELKLEDKHRTVIQRDVGHHVAYTTREVGQYLVVEASVGVIVIW
DKKTTVFIKLAPSYKGTVCGLCGNFDQRSNNDFTTRDHMVVDSELDFGNSWKEASACPDVSTIPVPCSLN
PHRRSWAEKQCSLIKSKVFRACHSKVDPKPFYEACVHDSCSCDTGGDCECFCSAVASYAQECTKEGACVF
WRTPDLCPIFCDYYNPPDECEWHYEPCGNRSFETCRTINGIHSNISVSYLEGCYPRCPKNRPIFDEDRKKCV
PADKCGCYLEDIHYPPGASVPPEHDCQACLCTASSQVVCWPEEGKIVNYTQDGSFCYWEICGPNGTRVP
HFNVCVSTPSTPTTSPTTALSPLSLCCVWSDWINDHHPSGDSDGGDHETFDHVCRAPQDIQCRAATEPH
LGWTELDQEAQCNISFGFICKNEDQFGKGPFGLCYDYEIRVYCCLPMEECPTTTPPPTPSSSTPTVPTTTPI
PPTTTPTPPTTTPTPPPLSTTSTTPPTTTPVPPTTSTTPPTTTPVPPTTTPTPPTTTPISTPPTTTSTTPPTTTPI
SPTTTPTPPTTTPTTTPLSTTSTTPPTTTPIPSTTSTTPPTTTPISTPPSTTSTTPPTTTPVPPTTSTTPPTTTLIST
PQSTTSTTPPTTTPIPTTTTTTPPTTTPTPSPPSTTSTTPPTTTPFPPTTSTTPPTTTPISTPPSTTSTTPPTTTPI
PPTTTPTPPTTTPTPTPPTTTSTPPTTTSTTPPPTSTLTSTPPPVSTTTVTPCLPQCKWTGWLDSGKPSFDKT
GGDFESLEDICELGWAADISCRAAMYPSVPIQELGQTVLCNTSVGLVCRNQDQKPGGAIPMPYCLNYEIN
VYCCELLDGCVSTTLTTTTSSTTTPITTLPTTTSTTPPTTTPIPTTSTTPPTTTPFPPTTSTTPPTTTPISTPPTPT
STTPPTTTPVPTTTTPAPTTTTPTVTPLSTTSTTPPTTTPFPPTTSTTPPTTTPTPSPPSTTSTTPPTTTPVPTTT
TPAPTTTTPTVTPLSTTSTTPPTTTPIPTTTTTTPTPSPPSTTSTTPPTTTPIPTTTTLTPPTTTPISTPPSTTSTTP
PTTTPIPTTTTTTPTPSPPSTTSTTPPTTTPFPPTPSTTPPTTTPISIPQSTTSTTPPTTTPIPPTTTHTPPTTTPIS
TPPTTTSTTPPTTTPISPTTTPTPPTTTPISTPLSTTSTSPPTTTPILPTTTPVPTTTTPTPSPPSTTFTTPPTTTSI
PPTTTPAPTTTTPISTLPTTISTPLTTTSTTLPPTSTSASMPPPVSTTTMTPPTPCLPQCKWTGWVDSDKPN

VNKTDGDFESLENICGLGWADDISCRAAMLPRIPIQELGQTVVCNTSVGLVCRNQDQKPVGASPTSYCL

NYEINVYCCDLPDYCVFTSSTTTAETPTTSPTTTSTETSSSTPTTTVTLTPTPTRTSTGTPTPASTTIETTVTET

PTPTPTTTSTGTPTPTPTTSTGTPTPSPTTTSTGIPTLTPTSTETTTTTTTTETPIPSPTTTSSETPTPTPTTTLTG

TPTPTPTTSTGTPTPTPTTTSTGTPTPTPTTSPATPTPTPTTTSTGTPTPTPTTTSTGTPTPTPTTTSTGTPTP

SPTTTSTGTPTPTPTTTSTGTPTPTPTTTSTGTPPPTPTTSTGTPTPTSTTTSTVTPTPTPTTSTGTPTPSPTTT

STGTPPPTPTTSTGTPTPTPTTSTGTPTPSPTTTSTGIPTLTPTSTETTTTTTTTETPIPSPTTTSSETPTPTPTT

TLTGTPTPTPTTSTATPTTTTTTTSTGTPTPSPTTTSTGTPTPTPTTTSTGTPTLTPTTSTGTPTPTPTTTSTGT

PTPTPTTSTGTPTPTPTTSTATPTTTTTTTSTGTPTPTPPTTSTGTPTPTSTTTSTVTPTPTPTTTSTGTPTPTP

TTTSTGTPPPTPTTSTGTPTPSPTTTSTGIPTLTPTSKETTTTTTTTETPIPPPTTTSTETPTPTPTTTLTGTPTP

TPTTSTGTPTTTPTTTSTGTPTPTPTTTSTGTPTPTPTTTSTGTPTPTRTTTSTGTPTPTPTTTSTGTPTPTPT

TTSTGTPPPTPTTSTGTPTPTPTTTSTETPTPSPTTTSTGIPTLTPTSKETTTTTTTTETPIPSPTTTSTETPTPTP

TTTLTGTPTPTPTTSTGTPTPTPTTSTGTPTPSPTTTSTGTPPPTPTTSTGTPTPTPTTTSTGTPTPSPTTTST

GIPTLTPTSTETTTTTTTTETPIPSPTTTSTETPTPTPTTTLTGTPTPTPTTTSTGTPTPTPTTTSTGTXXXXXXX

XXXXXXXXXXXXXXXXXXXXXXXXXXXXXXXXXXXTTSTGTPTPTPTTTSTGTPTPSPTSTSTGTPTPTPT

TTSTGTPTPSPTTTSTVTPTPTPTSTSTIVETFSTPLTPTSTEPVVTTTGGATSSSPTPGTTPPGTQAARSQCC

YRFFESLTWEPAWEAGCRAWVAAATSWGSGTWASVSWSLRGPAPHGWRQPQAPPAGELVYNGTHG

DTCYYVNCSLDCNLEFFNWSCPSTPTTQPPTTPSVPATSKPLPACPDFDPPRQENETWWLCNCTMATCK

YNNTVELVEVKCDPPPMPTCSNGLAPVRVMDPNGCCWHWECDCYCTGWGDPHYVTFDGLYYSYQGN

CTYVLVEEATPTVDNFGVYIDNYHCDVNDQVSCPRTLIVRHETQEVLLKTVHMMPVTVQVQVNRQVVA

LPYKKYGLQVYESGINYVVDIPDLGALISYNGLSFSVRLPYRLFGNNTKGQCGTCTNTTADDCVLPSGEVTD

DCEVAADQWVVNDPSKPHCPHISFTTRSPATSPPVGRGTTARKDCASPLCELIKDSLFAECHALAPPQHY

YEACMFDSCFVPNSGLECASLQTYAALCAQEGICVDWRNHTHGACPVTCPAHREYRACGPAEEPTCKSS

PSQQNSTRLVEGCFCPEGTMNYAPGFDVCVDFCGCVGPDNVPREFGEHFEFDCKHCLCLEGGSGITCRP

KTCAPEQRVECREDGTYPVREVDPLDTCCNVTSCKCNASLCREQPPLCPLGFQVNSQMVPGRCCPRYSC

EPKGVCVIDQAEYQPGSPVYSSKCQNCICTDRRDNATQLNVISCTHVPCNTSCNPGFELVDAPGECCKKC

QQTQCVINLPDNQKLVLKPGDRKRDSLDNCTFFSCVKAHDQLISSVSSITCPDFDPSTCLPGSVTLMRNGC

CRKCIPRNETRVACSTISVVREISYDGCTALVTMNDCSGSCGTFAMYSAQAQSLDHKCSCCKEYRTSQREV

TLQCPDGGTLSHTYTHIESCLCQDTVCQLPSQRRTRRSGPRVLGPGTG

2 SDS-PAGE with Coomassie staining

An SDS-PAGE was conducted with purified MUC5AC, purified MUC2, and MUC5B repurified from commercial BSM as described in the main text but with an increased sample loading volume of 25 µL. After the electrophoresis run, the gel was stained in a Coomassie Blue staining solution for 1.5 h under gentle shaking.

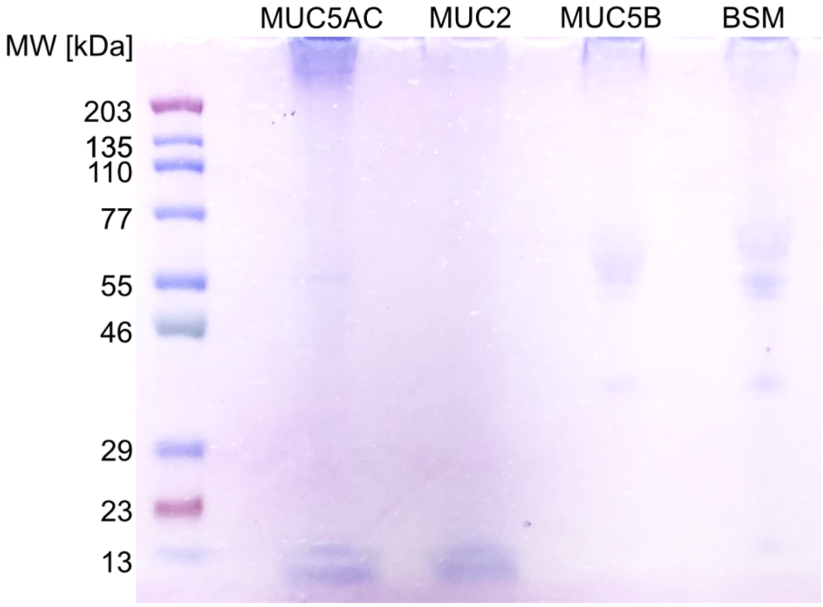

Fig. S1 *Purity of mucins.* SDS-PAGE of purified MUC5AC, MUC2 and MUC5B samples and off-the-shelf purity of commercial BSM stained with Coomassie staining.

3 Hydrodynamic size

Dynamic light scattering was employed to measure the hydrodynamic size of pure and mixed mucins. For the pure mucin samples, MUC5AC, MUC5B, and MUC2 were separately dissolved in Dulbecco`s Phosphate Buffered Saline (D-PBS) at a concentration of 0.05 % (w/v) and then measured using a Litesizer 500 (Anton-Paar, Graz, Austria) in a four-clear-sided disposable cuvette at a target temperature of 25°C and a detection angle of 90° (side scattering mode). The hydrodynamic diameter was automatically calculated by the device based on the assumption of a spherical particle shape. Mixed mucin samples were prepared by separately

dissolving MUC5AC, MUC5B, and MUC2 at a concentration of 0.1 % (w/v) in ultrapure water. Next, the concentration of these stock solutions was adjusted to 0.05 % (w/v) using 20 mM Sodium Acetate buffer at a desired pH (pH 3 or pH 4 - depending on the specific sample) and measured in the same way as the pure mucin samples. All samples were equilibrated for 2 h at room temperature before the measurement.

Contrary to observations made at neutral pH, at acidic pH MUC5AC and MUC5B tend to form polydisperse aggregates. This can most likely be attributed to the protonation of amino acids such as leucine, arginine, and histidine in the terminal regions of the mucin molecules. This protonation is considered to cause partial unfolding of the mucin termini, thereby exposing hydrophobic domains that eventually cause aggregation of the mucin molecules[4]. Interestingly, the aggregates formed in mixed mucin solutions at acidic pH tend to be smaller than those observed for the pure mucins."

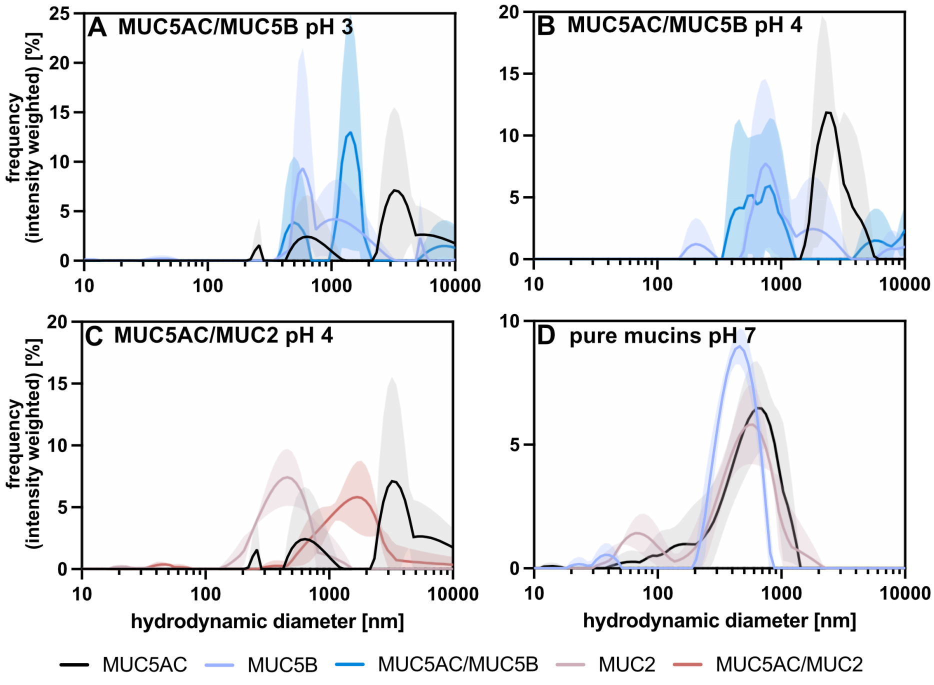

Fig. S2 *Hydrodynamic size of pure and mixed mucins.* Hydrodynamic diameters for binary mixtures of MUC5AC/MUC5B at pH 3 (A) and pH 4 (B) and MUC5AC/MUC2 at pH 3 (C) as well as pure mucins at pH 7 (D); shaded areas indicate the standard deviation (n = 3).

4 Turbidity of pure and mixed mucin samples over time

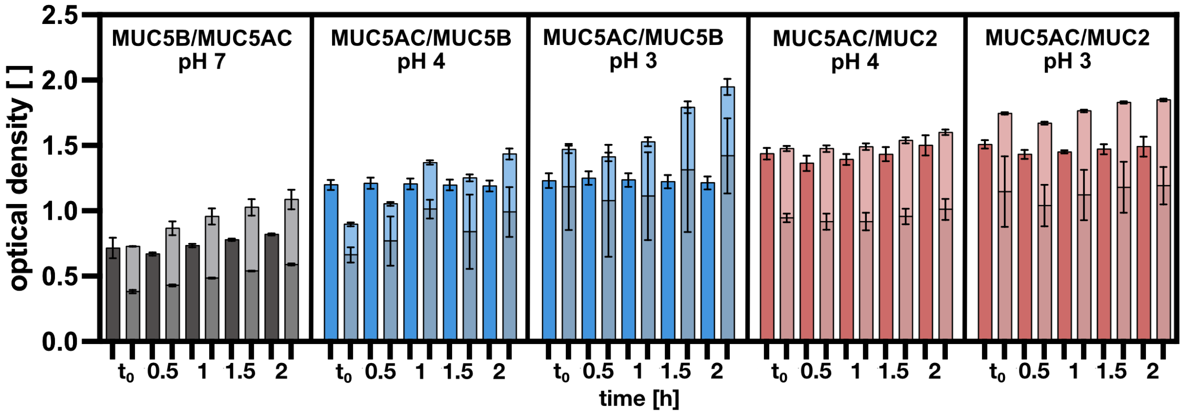

Fig. S3 *Turbidity of pure and mixed mucin samples over time*. Measured turbidity of binary mixtures of mucin subtypes (full bars) compared to their expected turbidity according to the sum of their individual components (stacked bars; lower bar: host system; upper bar: contaminating mucin subtype) recorded over time; error bars indicate the standard deviation (n = 3).

5 Turbidity of MUC5AC/MUC5B samples at different mixing ratios

Turbidimetric measurements of MUC5AC/MUC5B samples at pH 3 were conducted as described in the main text but with varying mixing ratios (MUC5AC/MUC5B: 65/35; 75/25; 85/15).

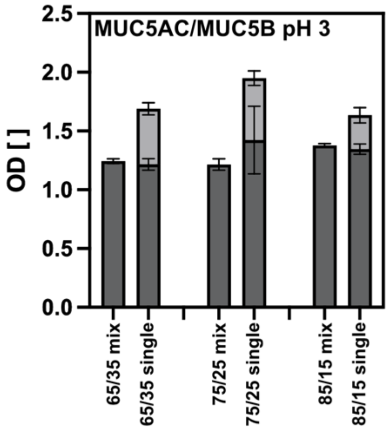

Fig. S4 *Turbidity of pure and mixed mucin samples*. Measured turbidity of binary mixtures of MUC5AC and MUC5B at different mixing ratios (full bars) compared to their expected turbidity according to the sum of their individual components (stacked bars; lower bar: MUC5AC; upper bar: MUC5B) after 2 h of equilibration; error bars indicate the standard deviation (n = 3).

6 Overlay structures

When predicting the folding structure of an amino acid sequence with AlphaFold 3, the software generates five individual structure predictions. Overlay images of these individual structure predictions for complexes comprising two and four N-termini (of different combinations of mucin variants), respectively, are depicted in **Fig. S5**. For all complexes, at least 4 out of 5 structure predictions were in good agreement with each other and thus were included in the overlay images. This indicates a high level of confidence of the prediction result.

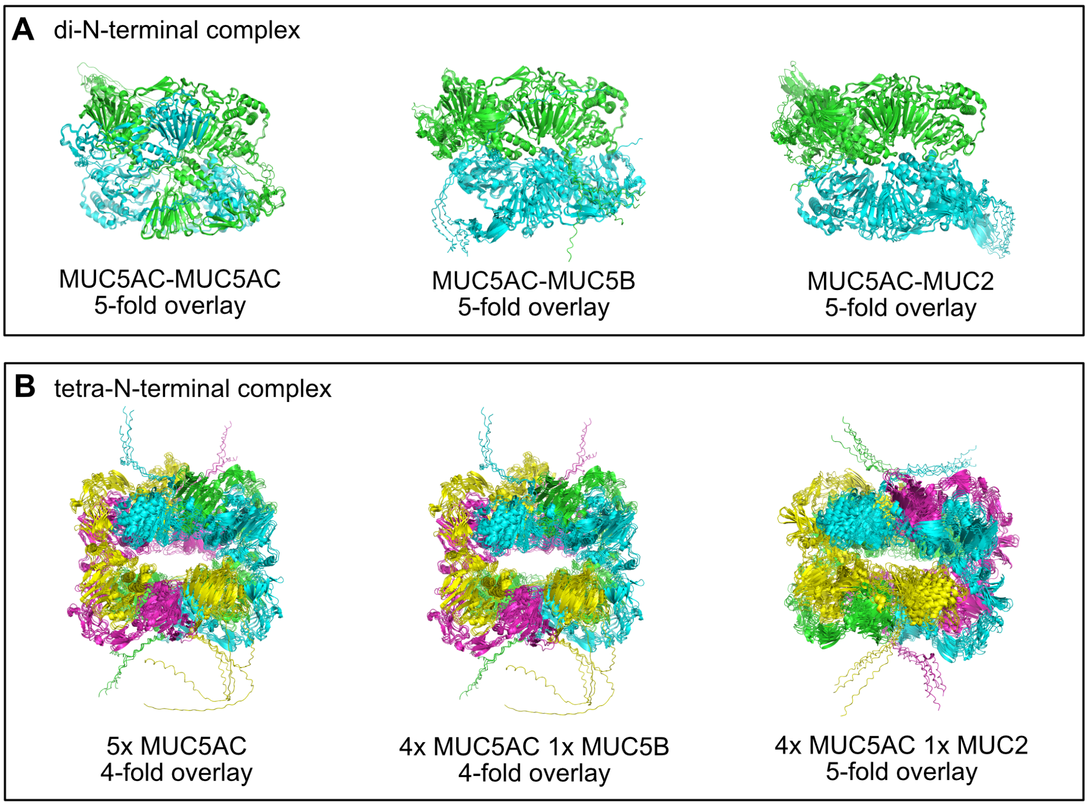

Fig. S5 *Overlay images of structure predictions.* Overlay images of complexes comprising two N-termini (A) or four N-termini (B). Each complex comprises one or more MUC5AC N-termini and one MUC5AC, MUC5B, or MUC2 N-terminus as an additional interaction partner. All complexes are color coded with respect to the chains of the individual N-terminus. The labels '4-fold overlay' and '5-fold overly' indicate how many structure predictions were included in the overlay image.

7 Alignment of amino acid sequences

The amino acid sequence of the N-terminal region of MUC5AC was aligned with the corresponding sequences of MUC5B and MUC2 using the Basic Local Alignment Search Tool for proteins (blastp suite, National Library of Medicine, Rockville Pike, USA). A pairwise comparison of the aligned sequences is shown below. Dots (“.”) indicate identical amino acids between the query sequence (MUC5AC) and the subject sequence (MUC5B or MUC2), whereas “-“ indicate missing amino acids in the respective sequence.

MUC5AC/MUC5B:

```
>MUC5B
Sequence ID: Query_1616032 Length: 1100
Range 1: 243 to 952

Score:1001 bits(2587), Expect:0.0,
Method:Compositional matrix adjust.,
Identities:473/716(66%), Positives:568/716(79%), Gaps:6/716(0%)

Query  390   PCPGTCSVLGGAHFSTFDEREYTVHGDCSYVLAKPCNSSAFTVLAELRRCGLTDSETCLK  449
Sbjct  243   .AADN.TDE..S.I..Y..KL.D.........T.V.AD..L.......K.....N.N...  302

Query  450   SLTLSLAGGQTVIVVKASGEVFVNQIYTQLPVSTANVMLFRPSTFFIIAQTQLGLQLDVQ  509
Sbjct  303   TV....N..D.TVQIQ.N.G....S..S...M.V.D.TV....S...LV..GPW...Q..  362

Query  510   LVPVMQVFVRLAPQLRGHTCGLCGNFNSIQADDFRTISGLVEGTAAAFANTWKTQAACPN  569
Sbjct  363   ...L....L..D.AY..QM........QN.......V..V..A.................  422

Query  570   IKNNFEDPCSLSVENEKYAQHWCSRLTDTHGPFARCHAAVNPSTYYSNCMFDTCNCEKSE  629
Sbjct  423   V..S............N.......L..RPA...SP..SVIS.GPFH...L..........  482

Query  630   DCMCAALSSYVRACAARGVLLSGWRDGVCTTPTATCPKSLTYRYHISTCQATCRARSDEG  689
Sbjct  483   ...........Q..................KYASS...TQS.AHVVDS..P...SL.-QP  541

Query  690   DATCSVSFVPVDGCTCSNDTFLDDTGKCVPATSCPCYYRGSVVPNGESLHDGGAVCTCTQ  749
Sbjct  542   .VS.D.A.......V.PRG.....A.TG...EA....L..T.LAP..VV..N.V..S.VS  601

Query  750   GTLACIGGHDPTPVCVPPMVYFDCRNATPGATGAGCQKSCHTLDMDCYSSQCVPGCVCPS  809
Sbjct  602   .R.S.L.ATEQSTG..A...FL..S..SAD.P..E.VR......V..F.TH..S.....V  661

Query  810   GLVASGEGGCIPASDCPCVHNEASYPAGQTIRVGCNTCTCKNRTWQCTDQPCLATCAVYG  869
Sbjct  662   ..LSD.S...VAEE....M....A.KP.EV.K.D......RG.R.E.S.R...G..VA..  721

Query  870   DGHYLTFDGQRYSFSGDCEYTLVQDHCSGNGSAQDGFRVITENIPCGTTGTTCSKMUCAC  929
Sbjct  722   ...F.....E..G.E.S.....A..Y.V.SDT.NGT..IV...V......V....-----  776

Query  930   AIKLFLGSDELKLSDGKVEVIETDPGQPPPFAIRQMGIYLVVDTDAGLVLLWDKKTSIFL  989
Sbjct  777   ...I....Y..I.HE.THR.LQRG..GDL.YRV.Y.....T.E.HG.V.VS..R...VII  836

Query  990   TLSPEFKGRVCGLCGNFDGNALNDFTTRSQSVVGDVLEFGNSWKFSPSCPDARAPKDPCT  1049
Sbjct  837   R.RH.Y...........D.............AS..................L..R....  896

Query  1050  ANPYRKSWAQKQCSIINSATFSACRAHVEPARYYEACVSDACACDTGGDCECFCTA  1105
Sbjct  897   T....R...................SQ.D.T..............S..........  952


Range 2: 38 to 252

Score:279 bits(713), Expect:6e-82,
Method:Compositional matrix adjust.,
Identities:127/215(59%), Positives:164/215(76%), Gaps:1/215(0%)

Query  13    PGDLLRKVTVHPPLRTTPVVRALNAAHGGQVCSTWGDFHYKTFDGDVFRFPGLCNYVFSA  72
Sbjct  38    .EMTA.HL.FI..ITVF.TMSP.......R..........................I...  97

Query  73    HCGAAYEDFNIQLRRGPAPNATAPSRVTMKLDGMVVELTKSSVLVNGRTIQLPFSQSGVL  132
Sbjct  98    ...S......L.....LLGSRPTITHIVLRSQ.L.L.VSNG...I..WREE..Y.RA.L.  157

Query  133   VEQSSSYVKVVARLGLVFLWNQDDSLLLELDAKYANKTCGLCGDFNGIPVFNEFLSHG-K  191
Sbjct  158   ..R..T...INI..M.T.M..GE..A.....P....Q..........LRAVS..YA.NTR  217

Query  192   LSPIEFGNLQKMDGPMEQCQDPVPESPVNCSTSSG  226
Sbjct  218   ...LQ......L...T......L.SPAADNC.DE.  252


Range 3: 634 to 1081

Score:186 bits(473), Expect:9e-52,
Method:Compositional matrix adjust.,
Identities:147/477(31%), Positives:208/477(43%), Gaps:54/477(11%)

Query  309   GSPCADTCSNLEHSQLCEDHCVAGCFCPKGMVLDDVGHTGCISVSECSCVYNGVTYAPGT  368
Sbjct  634   .AE.VRS.HT.DVDCF-ST...S..V..V.LLS.--.SG..VAEED.P.MH.EAA.K..E  690

Query  369   GYSTDCSSCTCSGGRWSCQEVPCPGTCSVLGGAHFSTFDEREYTVHGDCSYVLAK-----  423
Sbjct  691   VIKV..NT...R.R..E.SDR..L...VAY.DG..L...GER.GFE.S.E.T..QDYCVG  750

Query  424   -PCNSSAFTVLAELRRCGLTDSETCLKSLTLSLAGGQTVIVVKASGEVFVNQIYTQLPVS  482
Sbjct  751   SDTANGT.RIVT.NVP..T.-GV..S.AIKIF.-.SYEL.LHEGTHR.LQRGPGGD..YR  808

Query  483   TANVMLFRPSTFFIIAQTQLGLQLDVQLVPVMQVFVRLAPQLRGHTCGLCGNFNSIQADD  542
Sbjct  809   V------.YMGIYLTVE.HG.VV--.SWDRKTS.II..RHEYK.RV.......DDNALN.  860

Query  543   FRTISGLVEGTAAAFANTWKTQAACPNIKNNFEDPCSLSVENEKYAQHWCSRLTDTHGPF  602
Sbjct  861   .T.R.QS.ASDVLE.G.S..FSPS..DALAP-R...TTNPYRRSW..KQ..IINS--AT.  917

Query  603   ARCHAAVNPSTYYSNCMFDTCNCEKSED--CMCAALSSYVRACAARGVLLSGWRDGVCTT  660
Sbjct  918   SA.RSQ.D.TR..EA.VS.A.A.DSGG.CE.F.T.VAA.AQ..HEA..CV.-..-----.  971

Query  661   PTATCPKSLTY-------RYHISTCQATCRARSDEGDATCSVSFVPVDGCT----CSNDT  709
Sbjct  972   .D-V..LFCD.YNPHGQCEW.YQP.G.P.LKTCRNPSGL.LMDLPGLE..YPKCPS.KPF  1030

Query  710   FLDDTGKCVPATS------CPCYYRGSVVPNGESLHDGGAVCTCTQGTLACIGGHDP  760
Sbjct  1031  .NE.QME..AQC.GCYDGDGNY.DA.TR..ST.NCQS----.D..SSG.Q.T--.S.  1081
```

Range 4: 717 to 1100

Score:119 bits(298), Expect:8e-31,
Method:Compositional matrix adjust.,
Identities:110/403(27%), Positives:163/403(40%), Gaps:46/403(11%)

```
Query  44    CSTWGDFHYKTFDGDVFRFPGLCNYVFSA-HC---GAAYEDFNIQLRRGPAPNATAPSRV  99
Sbjct  717   .VAY..G.FL....ERYG.E.S.E.TLAQDY.VGSDT.NGT.R.VTENV.CGTTGVTCSK  776

Query  100   TMKL----DGMVVELTKSSVLVNGRTIQLPFSQSGVLVEQSSSYVKVVARLGLVFLWNQD  155
Sbjct  777   AI.IFLGSYELILHEGTHR..QR.PGGD..YR-----.RYMGI.LT.ETHG.V.VS.DRK  831

Query  156   DSLLLELDAKYANKTCGLCGDFNGIPVFNEFLSHGKL---SPIEFGNLQKMDGPMEQC--  210
Sbjct  832   T.VIIR.RHE.KGRV.....N.DD-NAL.D.TTRSQSVASDVL....SW.FSPSCPDALA  890

Query  211   -QDPVPESPVNCSTSSGICKEMLSELFPGCAALVDASSYLNACQHDLCRCQQANLTSCLC  269
Sbjct  891   PR..CTTN.YRR.WAQKQ.SIIN.AT.SA.RSQ..PTR.YE..VS.A.A.DSGGDCE.F.  950

Query  270   HTLAEYSRQCAHAGGQPLDWRGPHLCPQTCPQ-------NTEYRECGSPCADTCSNLEHS  322
Sbjct  951   TAV.A.AQA.-.EA.VCVS..T.DV..LF.DYYNPHGQCEWH.QP..A..LK..R.PSGL  1009

Query  323   QLCEDHCVAGCF--CPKGMVLDDVGHTGCISVSECSCVY--NGVTYAPGTGY--STDCSS  376
Sbjct  1010  C.MDLPGLE..YPK..SSKPFFNEDQME.--.AQ..GC.DGD.NY.DA..RVPSTEN.Q.  1067

Query  377   CTCSGGRWSCQEVPCPGTCSVLGGAHFSTFDEREYTVHGDCSY  419
Sbjct  1068  .D.TSSGLQ.THS.EAC..----------.YEG.T.-AY..VI.  1100
```

Range 5: 248 to 606

Score:106 bits(264), Expect:8e-27,
Method:Compositional matrix adjust.,
Identities:102/364(28%), Positives:148/364(40%), Gaps:26/364(7%)

```
Query  44   CSTWGDFHYKTFDGDVFRFPGLCNYVFSAHCGAAYEDFNIQLRR-GPAPNATAPSRVTMK  102
Sbjct  248  .TDE.GS.IS.Y.EKLYDVH.D.S..LTKV.ADSALTVLAE..KC.LTD.ENCLKT..LS  307

Query  103  LDG---MVVELTKSSVLVNGRTIQLPFSQSGVLVEQSSSYVKVVARLGLVFLWNQDDSLL  159
Sbjct  308  .N.GDTT.QIQANGG.F..SIYS...M.VAD.T.FRP..FFIL.QTGPWLQ.QV.LVP.M  367

Query  160  ---LELDAKYANKTCGLCGDFNGIPV--FNEFLSHGKLSPIEFGNLQKMDGP----MEQC  210
Sbjct  368  QVF.R..PA.RGQM.....N..QNQADD.RTVSGVVEATAAA.A.TW.TQAACPNVKNSF  427

Query  211  QDPVPESPVNCSTSSGICKEMLSEL--FPGCAALVDASSYLNACQHDLCRCQQANLTSCL  268
Sbjct  428  E..CSL.VE.ENYAQHW.SLLTRPAGP.SP.HSVISPGPFHSN.LF.T.N.EKSE--D.M  485

Query  269  CHTLAEYSRQCAHAGGQPLDWRGPHLCPQ---TCPQNTEYRECGSPCADTCSNLEHSQLC  325
Sbjct  486  .AA.SS.VQA..AR.VLLSG..D-GV.TKYASS..KTQS.AHVVDS.QP..RS.SQPDVS  544

Query  326  EDHC---VAGCFCPKGMVLDDVGHTGCISVSECSCVYNGVTYAPGTGYSTDCSSCTCSGG  382
Sbjct  545  C.VAFVP.D..V..R.TF...A.-..-VPAEA.P.YLR.TVL...EVVHDNGVV.S.VS.  602

Query  383  RWSC  386
Sbjct  603  .L..  606
```

Range 6: 68 to 268

Score:73.2 bits(178), Expect:1e-16,
Method:Compositional matrix adjust.,
Identities:54/210(26%), Positives:92/210(43%), Gaps:12/210(5%)

```
Query  394  TCSVLGGAHFSTFDEREYTVHGDCSYVLAKPCNSSAFTVLAELRRCGLTDSETCLKSLTL  453
Sbjct  68   V..TW.DF.YK...GDVFRFP.L.N.IFSAH.G.AYEDFNLQ...-..LG.RPTITHIV.  126

Query  454  SLAGGQTVIVVKASGEVFVNQIYTQLPVSTANVMLFRPSTFFIIAQTQLGLQLDVQLVPV  513
Sbjct  127  R---S.GLVLEVSN.S.LI.GWREE..Y.R.GLLVE.S..YVK.---NIR.M.TFMWNGE  180

Query  514  MQVFVRLAPQLRGHTCGLCGNFNSIQA-DDFRTISGLVEGTAAAFANTWKTQAACPNIKN  572
Sbjct  181  DSALLE.D.KYANQ......D..GLR.VSE.YAHNTRL--SPLQ.G.LQ.LDGPTEQCQD  238

Query  573  NFEDPCSLSVENE--KYAQHWCSRLTDTHG  600
Sbjct  239  PLPS.AADNCTD.GGSHISTYDEK.Y.V..  268
```

Range 7: 68 to 229

Score:70.9 bits(172), Expect:6e-16,
Method:Compositional matrix adjust.,
Identities:45/172(26%), Positives:82/172(47%), Gaps:11/172(6%)

```
Query  864   TCAVYGDGHYLTFDGQRYSFSGDCEYTLVQDHCSGNGSAQDGFRVITENIPCGTTGTTCS  923
Sbjct  68    V.STW..F..K....DVFR.P.L.N.-IFSA..---...YED.NLQLRRGLL.SRP.ITH  123

Query  924   KMUCACAIKLFLGSDELKLSDGKVEVIETDPGQPPPFAIRQMGIYLVVDTDAGLVLLWDK  983
Sbjct  124   IVLRSQGLV.EVSNGSVLINGWRE.LPYSRA.----LLVERSST.VKINIRLM.TFM.NG  179

Query  984   KTSIFLTLSPEFKGRVCGLCGNFDG-NALNDFTTRSQSVVGDVLEFGNSWKF  1034
Sbjct  180   ED.AL.E.D.KYANQT.....D.N.LR.VSE.YAHNTRL--SP.Q...LQ.L  229
```

MUC5AC/MUC2:

```
>MUC2
Sequence ID: Query_6305083 Length: 1100
Range 1: 32 to 1094

Score:1123 bits(2906), Expect:0.0,
Method:Compositional matrix adjust.,
Identities:536/1067(50%), Positives:713/1067(66%), Gaps:9/1067(0%)

Query  39    HGGQVCSTWGDFHYKTFDGDVFRFPGLCNYVFSAHCGAAYEDFNIQLRRGPAPNATAP--  96
Sbjct  32    ..HN........................D.N.ASD.RD..KE.AVH.....GGSGGPSQV  91

Query  97    SRVTMKLDGMVVELTKSSVLVNGRTIQLPFSQSGVLVEQSSSYVKVVARLGLVFLWNQDD  156
Sbjct  92    EYILLTVKDDTIY..QQL.V...AMVST.HYSP.L.I.R.AI.T..YS.A..ALV..RE.  151

Query  157   SLLLELDAKYANKTCGLCGDFNGIPVFNEFLSHG-KLSPIEFGNLQKMDGPMEQCQDPVP  215
Sbjct  152   .VM....S.FQ.H.......Y..LQTYS....E.IPF..L....M..INK.E.K.D..EE  211

Query  216   -ESPVNCSTSSGICKEMLSEL-FPGCAALVDASSYLNACQHDLCRCQQANLTSCLCHTLA  273
Sbjct  212   AQAKLS..EHRAE.ERL.TDVA.ED.QG..PLEL.VQ..VQ.R.Q.P.G--...V.S.I.  269

Query  274   EYSRQCAHAGGQPLDWRGPHLCPQTCPQNTEYRECGSPCADTCSNLEHSQLCEDHCVAGC  333
Sbjct  270   .F....S....R.GN..TAT...KS..G.MV.L.SS...V....H..V.S...E.RMD..  329

Query  334   FCPKGMVLDDVGHTGCISVSECSCVYNGVTYAPGTGYSTDCSSCTCSGGRWSCQEVPCPG  393
Sbjct  330   ...E.T.Y..IAGR...P..Q.H.KLH.HQ....QQVTNN.EQ.V.NA...V..DLQ...  389

Query  394   TCSVLGGAHFSTFDEREYTVHGDCSYVLAKPCNSSAFTVLAELRRCGLTDSETCLKSLTL  453
Sbjct  390   A.ALE..S.IT...G.K..F....Y...T.GTHNDSYAI.G..TP..S..KQ....TVV.  449

Query  454   SLAGGQTVIVVKASGEVFVNQIYTQLPVSTANVMLFRPSTFFIIAQTQLGLQLDVQLVPV  513
Sbjct  450   LADNKKN.VLF.SD.S.LL.ELQVN..HV..SFSI.Q..SHHLLVD.AF..R.Q...A.M  509

Query  514   MQVFVRLAPQLRGHTCGLCGNFNSIQADDFRTISGLVEGTAAAFANTWKTQAACPNIKNN  573
Sbjct  510   ..L.LT.DQAAQ.RVQ.......GLEG...K.AG....A.G.G......A.SS.HDKVDW  569

Query  574   FEDPCSLSVENEKYAQHWCSRLTDTHGPFARCHAAVNPSTYYSNCMFDTCNCEKSEDCMC  633
Sbjct  570   LD.....NI.SAN..E....L.KK.GT..G...S..D.AE..KR.KY.....QNT...L.  629

Query  634   AALSSYVRACAARGVLLSGWRDGVCTTPTATCPKSLTYRYHISTCQATCRARSDEGDATC  693
Sbjct  630   ......A.....K.IM.W...EH..NKDVGS..Q.QIF..NLT...Q...SL.-.A..H.  688

Query  694   SVSFVPVDGCTCSNDTFLDDTGKCVPATSCPCYYRGSVVPNGESLHDGGAVCTCTQGTLA  753
Sbjct  689   LEG.A.....G.PDQ....EK.R...LAK.S..H..LYLEA..VVLRQEER.V.RS.R.H  748

Query  754   CIGGHDPTPVCVPPMVYFDCRNATPGATGAGCQKSCHTLDMDCYSSQCVPGCVCPSGLVA  813
Sbjct  749   .VPVKLLGQS.EA.KIHV..N.L.AL.IRNPRPV..Q..AAGY.QTE..S.....E..ID  808

Query  814   SGEGGCIPASDCPCVHNEASYPAGQTIRVGCNTCTCKNRTWQCTDQPCLATCAVYGDGHY  873
Sbjct  809   D.R...VVEEA.......DL.SP.DR...D......RKGR.A..QSV.HG...I..S...  868

Query  874   LTFDGQRYSFSGDCEYTLVQDHCSGNGSAQDGFRVITENIPCGTTGTTCSKAIKLFLGSD  933
Sbjct  869   I....KY.D.D.H.S.VA...Y.-.QK.P.GS.SI....V......V.......I...RT  927

Query  934   ELKLSDGKVEVIETDPGQPPPFAIRQMGIYLVVDTDAGLVLLWDKKTSIFLTLSPEFKGR  993
Sbjct  928   ....E.KHRT..QR.V.HHVAYTT.EV.Q....EASV.VIVI.....TV.IK.A.SY..T  987

Query  994   VCGLCGNFDGNALNDFTTRSQSVVGDVLEFGNSWKFSPSCPDARAPKDPCTANPYRKSWA  1053
Sbjct  988   .........QRSN......DHM..DSE.D......EASA...VSTIPV..SL..H.R...  1047

Query  1054  QKQCSIINSATFSACRAHVEPARYYEACVSDACACDTGGDCECFCTA  1100
Sbjct  1048  E....L.K.KV.R..HSK.D.KPF.....H.S.S...........S.  1094


Range 2: 793 to 1100

Score:180 bits(456), Expect:8e-50,
Method:Compositional matrix adjust.,
Identities:105/323(33%), Positives:167/323(51%), Gaps:22/323(6%)

Query  326   EDHCVAGCFCPKGMVLDDVGHTGCISVSECSCVYNGVTYAPGTGYSTDCSSCTCSGGRWS  385
Sbjct  793   QTE..S..V..E.LI--.D.RG..VVEEA.P..H.EDL.S..DRIRV..NT...RK...A  850

Query  386   CQEVPCPGTCSVLGGAHFSTFDEREYTVHGDCSYVLAK-----PCNSSAFTVLAELRRCG  440
Sbjct  851   .TQSV.H...AIY.SG.YI...GKY.DFD.H....AVQDYCGQKSPQGS.SIIT.NVP..  910

Query  441   LTDSETCLKSLTLSLAGGQTVIVVKASGEVFVNQIYTQLPVSTANVMLFRPSTFFIIAQT  500
Sbjct  911   T.-GV..S.AIKIF.--.R.ELKLEDKHRTVI-----.RD.GHHVAYTT.EVGQYLVVEA  962

Query  501   QLGLQLDVQLVPVMQVFVRLAPQLRGHTCGLCGNFNSIQADDFRTISGLVEGTAAAFANT  560
Sbjct  963   SV.VI--.IWDKKTT..IK...SYK.TV.......DQRSNN..T.RDHM.VDSELD.G.S  1020

Query  561   WKTQAACPNIKNNFEDPCSLSVENEKYAQHWCSRLTDTHGPFARCHAAVNPSTYYSNCMF  620
Sbjct  1021  ..EAS...DV-STIPV....NPHRRSW.EKQ..LIKSK--V.RA..SK.D.KPF.EA.VH  1077

Query  621   DTCNCEKSEDC--MCAALSSYVR  641
Sbjct  1078  .S.S.DTGG..ECF.S.VA..AQ  1100


Range 3: 812 to 1100

Score:87.4 bits(215), Expect:5e-21,
Method:Compositional matrix adjust.,
Identities:82/298(28%), Positives:125/298(41%), Gaps:30/298(10%)

Query  1     GGCVSPALFP-------VSPGDLLRKVTVHPPLRTTPVVRALNAAHGGQVCSTWGDFHYK  53
Sbjct  812   ....VEEAC.CVHNEDLY....RI.VDCNTCTC.KGRWACTQSVC..--T.AIY.SG..I  869

Query  54    TFDGDVFRFPGLCNYV-FSAHCG--AAYEDFNIQLRRGP--APNATAPSRVTMKLDGMVV  108
Sbjct  870   ....KYYD.D.H.S..AVQDY..QKSPQGS.S.ITENV.CGTTGV.CSKAIKIF.GRTEL  929

Query  109   ELTKSSVLVNGRTIQLPFSQSGVLVEQSSSYVKVVARLGLVFLWNQDDSLLLELDAKYAN  168
Sbjct  930   K.EDKHRT.IQ.DVGHHVAYTTRE.G.---.LV.E.SV.VIVI.DKKTTVFIK.APS.KG  986

Query  169   KTCGLCGDFNGIPVFNEFLSHGKL---SPIEFGNLQKMDGPMEQCQD----PVPES--PV  219
Sbjct  987   TV.....N.DQRSN-.D.TTRDHMVVD.ELD...SW.---EASA.P.VSTI...C.LN.H  1042

Query  220   NCSTSSGICKEMLSELFPGCAALVDASSYLNACQHDLCRCQQANLTSCLCHTLAEYSR  277
Sbjct  1043  RR.WAEKQ.SLIK.KV.RA.HSK..PKPFYE..V..S.S.DTGGDCE.F.SAV.S.AQ  1100
```

Cited Literature

(1) Mucin-5AC A0A8W4FM62_PIG. https://www.uniprot.org/uniprotkb/A0A8W4FM62/entry (accessed November, 8 2024).
(2) Mucin-5B F2FB42_BOVIN. https://www.uniprot.org/uniprotkb/F2FB42/entry (accessed November 8, 2024).
(3) Muicn-2 A0A8D1ICB6_PIG. https://www.uniprot.org/uniprotkb/A0A8D1ICB6/entry (accessed November 8, 2024).
(4) Bansil, R.; Turner, B. S. Mucin structure, aggregation, physiological functions and biomedical applications. *Curr. Opin. Colloid Interface Sci.* **2006**, *11* (2-3), 164-170. DOI: 10.1016/j.cocis.2005.11.001.
